# Patience is a virtue: lessons from a participatory approach to contextually tailor and co-create an employee wellness intervention for community health educators

**DOI:** 10.3389/fpubh.2025.1634264

**Published:** 2025-09-19

**Authors:** Mary C. Frazier, Megan J. Pullin, Samantha M. Harden

**Affiliations:** ^1^Translational Biology, Medicine, and Health, Virginia Tech, Roanoke, VA, United States; ^2^Department of Human Nutrition, Foods, and Exercise, Blacksburg, VA, United States

**Keywords:** co-creation, flourishing, wellbeing, holistic, yoga, adaptation, implementation, dissemination

## Abstract

**Introduction:**

Community health educators (CHE) translate empirical health evidence into actionable information to improve the health and wellbeing of communities, including underserved populations. However, the wellbeing of CHE themselves is threatened by chronic work-related stress. One understudied CHE cohort are employees of the federal Cooperative Extension System (herein: Cooperative Extension). The objective of this present study was to co-create a wellness intervention that is feasible and acceptable to CHE of Cooperative Extension.

**Methods:**

Applying a co-creation method, we first gathered formative data from an ongoing integrated research-practice partnership (IRPP) with CHE of Cooperative Extension to guide adaptations on intervention content, dose, and delivery. IRPP members shared key intervention considerations which informed a sequential exploratory mixed-methods approach. To garner contextual considerations and phenomena, we conducted four focus group sessions with CHE from nine different states (*N*=21, *n*=4 to 6 per session). We built a follow up survey based on qualitative findings to inform intervention delivery.

**Results:**

Members of the IRPP preferred holistic wellbeing, i.e., flourishing, as a comprehensive target for a CHE wellness intervention. Eighty-one percent (*n*=17) of focus group participants (90% Female, 62% White) completed the follow up survey. Focus group findings demonstrated a desire for a multi-component intervention (e.g., education, accessible group yoga practices) to address the multiple domains of flourishing and provided guidance on imagery and messaging of recruitment materials. Notably, participants emphasized scheduling as the greatest barrier to overcome. One participant shared that “I think there are probably solutions for this, but it may take a lot of patience while figuring it out.” Survey data elucidated intervention delivery preferences including timing for the intervention (47% preferring a Jan-Mar launch), time of day (early morning ranked highest); facilitator (52% yoga teachers, 24% peer CHE, 0% administrators); as well as the order of content delivery in intervention sessions.

**Discussion:**

Data from co-creation methods with CHE captured often overlooked nuance important for implementation, particularly tailoring the timing of intervention delivery. Beginning with the end in mind and taking careful consideration of contextual factors may improve feasibility and acceptability of intervention characteristics and ultimately increase reach, representativeness, and efficacy.

## Introduction

Lay health educators, agents, promotoras, and community health workers of the United States, have the passion and skills to deliver public health interventions as trusted individuals in their communities (1–3). In fact, prior to administrative funding changes in 2025, the National Institute of Health funded over 2,000 projects related to community health workers and educators in 2024 alone (4). However, these community health educators (CHE), especially those from or working with historically underserved communities, represent a target population themselves. CHE face chronic work-related stress and burnout from their people-oriented and emotionally charged work (5). The personal and professional health of CHE must be attended to, or they will not be able to effectively translate empirical health evidence into actionable information to address the most disparate health threats (e.g., cardiovascular disease, diabetes, and health inequity) (6–9), especially in underserved communities (10–12).

According to extant literature, workplace wellness interventions continue to demonstrate small (13) to modest (14) effects in terms of mental health, sedentary behavior, weight management, and self-reported well-being. The lack of large, robust impacts is, in part, due to heterogeneity in modality (e.g., digital versus in person), varied theoretical underpinnings, and which core elements of the intervention are included [e.g., behavior change techniques (15)]. That is, there is no one-size-fits-all workplace wellness promotion intervention—especially not when considering the variety of workplace types and demands. In the case of CHE, they, too, are not a monolith. However, because CHE often work in dispersed, nontraditional workplaces, there is a general need for accessible, adaptable, and equitable practices that can be implemented in a variety of settings and activities—such as programs offered synchronously or in a self-paced format (16). Virtual interventions are particularly promising for improving CHE health and wellbeing (17). Specifically, meta-analyses of virtual workplace wellness interventions tout improvements in psychological well-being and work effectiveness (18). While virtual worksite wellness interventions have predominantly focused on work productivity, psychological (e.g., anxiety, stress, burnout) and anthropometric (e.g., weight, blood pressure) measures (18, 19), holistic measures that align intervention outcomes with outputs that participants value most (20) may improve intervention uptake.

For millennia, scholars such as Aristotle have been promoting holistic wellbeing or “flourishing” (21). More recently, psychology and positive health researchers have advocated for a shift toward promotion of wellbeing instead of prevention of disease (22–25). Interestingly, a recent randomized controlled trial (26) demonstrated large effects on self-reported outcomes and preliminary data to support compliance with more objective measures (i.e., saliva) in a “happiness intervention” group (i.e., a 7 week intervention focused on promoting joy across various domains of community, work, pleasure, bliss). Essentially, this positive psychology approach to workplace wellness is on the rise (26) and may have more robust outcomes, even in a short protocol period. Flourishing has emerged as a psychometrically validated holistic wellbeing measure across cultures and worksites (20, 27, 28), encompassing outputs such as whether a person is happy, has fulfilling relationships, and feels that they are a “good” person. The promotion of flourishing aligns with the philosophy, art, practice, and science of yoga (29–32). Yoga is a biopsychosocial-spiritual system that originated from India based in ancient and modern yoga principles including mental, physical, and breath practices (33–36). Yoga principles for public health have positively impacted myriad populations and outcomes and align with scientific rigor across fields including physiology, psychology, and neuroscience (37–40).

In alignment with scientific evidence on yoga, CHE of one state system in Cooperative Extension perceived personal yoga practices as beneficial for improved relaxation, overall wellness, mental health, physical health, and self-care (41). In this prior survey-based participatory work, CHE also expressed interest in yoga practices not only for themselves but also for the communities they serve. Additionally, prior phases of a 9-week virtual wellbeing program for CHE of Virginia Cooperative Extension demonstrated improved CHE flourishing and promising feasibility and acceptability of group yoga-based programs in CHE settings (42, 43). However, these prior phases had high scheduling burden on participants. In other words, while previous wellbeing program core functions (i.e., the key ingredients or purposes of the intervention) are promising, the program forms (i.e., the strategies to bring about core functions) are suboptimal (44–46). Therefore, a gap persists in the implementation of an employee wellness intervention for CHE of Cooperative Extension. Using co-creation and mixed methods with participatory community implementation strategies (47, 48) at the pre-implementation phase to adapt existing core functions of a wellness program for CHE has potential to enrich the translation of yoga principles. The aim of this work is not only so that CHE flourish, but that, through their lived experiences, they and the people they serve live long, healthy, and purpose-filled lives (49, 50). Thus, the primary objective of this pre-implementation study was to co-create a tailored wellness intervention that is feasible and acceptable to CHE of the federal Cooperative Extension System.

## Materials and methods

### Research design overview

An existing and ongoing integrated research-practice partnership (IRPP) (51, 52) with CHE of one particular state system of Cooperative Extension served in an advisory capacity to co-create program content, dose, and delivery. IRPP members are from across each district of the state (100% female, average age 44, age range 25–65 year; 50% with 8 + years working in Cooperative Extension). This included, but was not limited to, language suggestions (e.g., do not describe program as “self-care”) as well as potential scheduling of the program. When asked about workplace wellness interventions, IRPP members expressed the potential ease of adapting previous wellbeing programs for CHE (41–43). Members of the IRPP did not want a program that focused only on one health behavior or one component of overall health (e.g., only relaxation practice or only education on physical activity practices). Instead, members of the IRPP preferred holistic wellbeing, i.e., flourishing, as a comprehensive target for a CHE wellness intervention and expressed interest in multiple yoga principles (movement, breath, and mindfulness) for both personal practice and community translation. This IRPP advisory discussion informed our exploratory mixed-methods (i.e., qualitative followed by quantitative) approach of inviting CHE from a national sample to participate in a focus group and then a follow-up survey. The IRPP members, focus group participants, and follow-up survey participants completed research activities and were not provided additional compensation. This study was IRB exempt as it was considered quality assessment.

### Intervention description

The Flourishing in Extension (FLEX) program is a virtual 9-week work-based wellness program with weekly asynchronous emailed newsletters and weekly synchronous 60-min sessions. FLEX is comprised of adapted core functions from prior phases (42, 43) that include journal reflections (53–55), behavior change techniques (e.g., group-dynamics, social support, goal setting, self-monitoring, feedback, and education) (15, 56), experiential learning (57), and yoga principles of meditation and moment-to-moment awareness (*dhyana* and *smrti/sati*), breathwork (*pranayama*), and postures (*asana*) (29, 35) (See Supplementary file 1 for initial week-by-week guide). The intervention is designed to target flourishing in CHE and to also provide skills in translating intervention components for downstream flourishing of CHE workplaces and communities (42, 43, 58).

### Focus groups

After initial design and adaptations of the FLEX program, we invited a purposive sample of CHE of different state Cooperative Extension Systems. State specialists distributed emails to CHE with information for pre-scheduled focus groups using a Qualtrics sign up form (Qualtrics, Provo, UT, May 2024). Aside from questions on sample characteristics, the sign-up form included validated metrics on flourishing (27) and burnout (59). Focus groups were facilitated by a female doctoral candidate and graduate research assistant (MCF) who was trained in qualitative data collection. The facilitator created brief participant engagement-related field notes after each focus group session. No previous partnership or research collaboration existed with invited CHE except for those who might have been previously trained to deliver a community-based program in Virginia, Pennsylvania, and North Carolina. Focus group participants were aware that the facilitator (MCF) was working to adapt the FLEX intervention based on their input. No specific characteristics of the facilitator were reported to participants. Focus groups were 60 minutes in duration, conducted and audio-recorded on Zoom, consisted of only the facilitator and focus group participants, and included prompts on wellbeing, flourishing, yoga principles, as well as ‘think-aloud’ sessions on samples of recruitment and program materials (60, 61). Prompts were not previously pilot tested, and no focus group session was repeated. We used Zoom software to auto-generate the focus group transcripts. Two researchers (MCF and MJP) reviewed and edited focus group transcripts to match audio files. Transcripts were not returned to participants for comment or correction. Although we did not seek data saturation *a priori* as part of this pre-implementation study, our sample size and data saturation are consistent with prior findings (62, 63). Opt-in consent was presented and obtained at the start of the sign up form and verbal consent was obtained at the start of each focus group. We used the COnsolidated criteria for REporting Qualitative research (COREQ) checklist for focus group reporting (64).

### Follow-up survey

Focus group input informed a follow up survey for gathering clarifying information to guide adaptations of intervention delivery and materials. We emailed the CHE who participated in the focus groups with a Qualtrics follow-up survey link. The follow-up survey included questions on program delivery (time of year, day of the week, national scheduling, start time, and program facilitator), program materials (weekly session order-of-events, colors), interest in pilot program, and preference on pilot start date. Survey participants also had an open-ended option to leave comments at the end of the survey. Participants completed surveys with median time of 6 min. Opt-in consent was presented and obtained at the start of the follow-up survey.

### Data analysis

We analyzed quantitative data for descriptive purposes only. For the follow-up survey, we calculated ranking scores by weighing each ranking level for each survey item option. For the qualitative data, we used an inductive approach and thematic analysis (65). Two researchers, both of whom have previously worked with Cooperative Extension CHE, were familiar with flourishing theoretical underpinnings (26, 28), and are registered yoga teachers (MCF and MJP), used Excel Microsoft to independently code the qualitative data from the focus groups before meeting to resolve discrepancies. A third reviewer with similar experience, familiarity, and yoga training (SMH) provided final resolution on unresolved discrepancies as needed. We determined major emergent themes, subthemes, and categories by quantitatively examining the data by number of focus groups and MU for each code using Microsoft Excel. Ultimately, this led to the following thresholds for major emergent codes: (1) spanned three or more of the focus groups (≥75% of focus groups), or (2) included five or more meaning units (MU) in at least two focus groups (≥5 MU and ≥50%). Focus group participants did not provide additional feedback after qualitative analysis.

### Cocreated intervention updates

We co-created intervention updates by integrating focus group “think aloud” input and follow up survey data to guide decision-making for intervention materials and factors. For the focus group input, two independent researchers (MJP and MCF) wrote notes on their reflections on CHE focus group data related to the recruitment materials in Microsoft Excel. Researchers met to discuss notes and grouped data into actionable updates to materials. The two researchers, who each have yoga teacher training and experience in yoga principles for public health and health equity, independently created mock-up recruitment materials that met all focus group input on recruitment materials. Mockups and notated data were presented to the primary investigator (SMH) before a qualitative data-informed decision was collectively reached.

## Results

### Sample characteristics

Of the 222 CHE whom we emailed the focus group sign-up form, 30 emails bounced, and 30 CHE signed up to participate in the four focus group sessions (16%). Four participants later declined (3 had scheduling conflicts, 1 unspecified). Of the remaining 26, 21 CHE participated in the focus group sessions (81% of initial interested, 4 to 6 CHE per session, 76% with no previous research collaboration, see Table 1 for full sample characteristics). Of the 21 CHE whom we emailed, 17 CHE completed the follow-up survey (81% of focus group participants). Focus group participants had an average burnout score of 4.3 of 8 (standard deviation: 1.1, low score indicates no burnout whereas high score indicates complete burnout) and had average flourishing scores of 7.3 of 10 (standard deviation: 1.0, high scores indicate greater flourishing). Follow-up survey participants had an average burnout score of 3.78 (standard deviation: 1.06).

**Table 1:** Sample characteristics of focus groups and follow-up survey participants.

Characteristic	Focus groups	Follow-up survey
Dropped	9 of 30	0 of 18
Remaining Total	21	18
	n (%)	n (%)
Gender		
Female	19 (90%)	15 (88%)
Male	0 (0%)	0 (0%)
Prefer not to say	2 (10%)	2 (12%)
Ethnicity		
European / White American	13 (62%)	11 (65%)
African / Black American	4 (19%)	3 (18%)
American Indian / Alaska Native American	1 (5%)	0 (0%)
Prefer not to answer	3 (14%)	3 (18%)
State		
Arizona	1 (5%)	0 (0%)
Georgia	5 (24%)	4 (24%)
Idaho	2 (10%)	2 (12%)
Iowa	1 (5%)	1 (6%)
Mississippi	1 (5%)	1 (6%)
North Carolina	5 (24%)	3 (18%)
Pennsylvania	2 (10%)	2 (12%)
Virginia	3 (14%)	3 (18%)
West Virginia	1 (5%)	1 (6%)
Cooperative Extension Area		
Agriculture	1 (5%)	0 (0%)
Family and Consumer Science	14 (67%)	11 (65%)
Family and Consumer Science, Community Viability	1 (5%)	1 (6%)
4H, Family and Consumer Science	3 (14%)	2 (12%)
Prefer not to answer	2 (10%)	2 (12%)

### Focus group findings

Transcripts from the focus group sessions resulted in 389 MU total. We present the data in three parts: (1) perspectives on wellbeing, flourishing, yoga principles, and wellbeing program (169 MU subtotal); (2) program materials input (125 MU subtotal); and (3) recruitment materials input (90 MU subtotal). For qualitative analysis, inter-rater reliability between the two coding researchers was 99%.

#### Perspectives on wellbeing, flourishing, yoga principles, and wellbeing program

**Figure 1:**
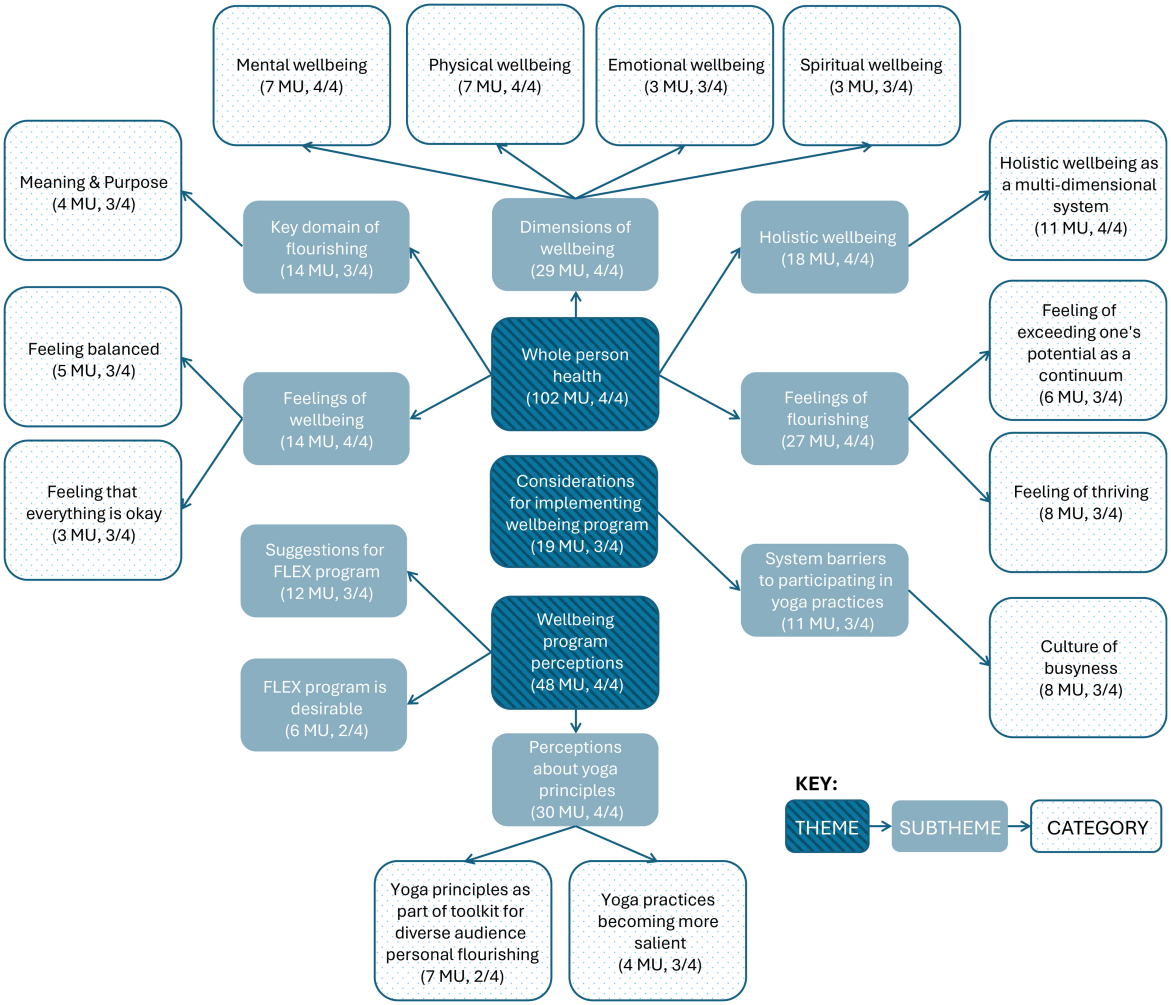
Major emergent themes, subthemes, and categories of focus group data on perspectives of wellbeing, flourishing, yoga principles, and wellbeing program. MU, meaning units. FLEX, Flourishing in Extension. Number of focus groups indicated by ‘n’/4.

**Figure 2:**
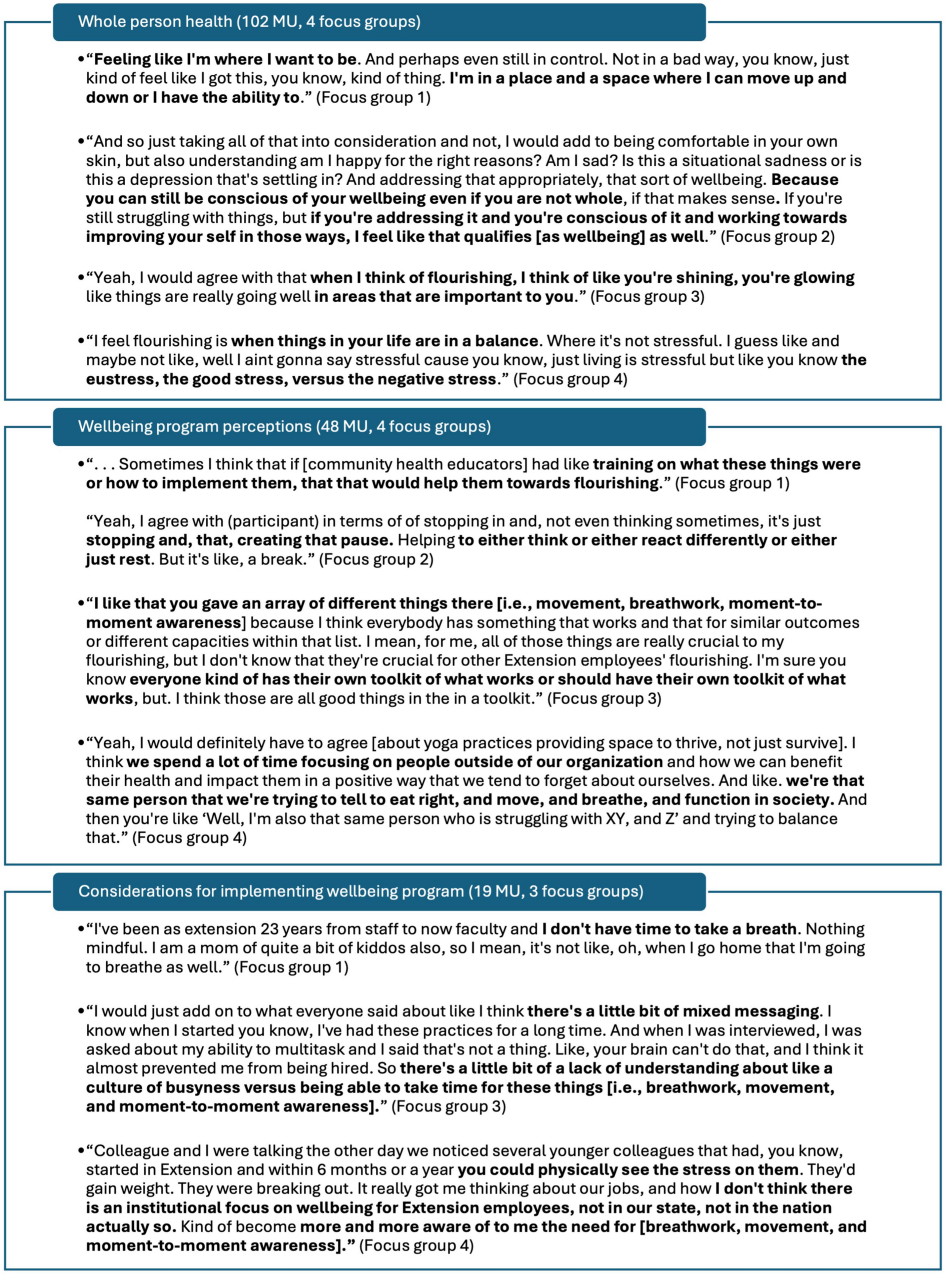
Exemplar meaning units under *whole person health, wellbeing program perceptions, and considerations for implementing wellbeing program* themes. MU, meaning unit.

Focus group participants described their perspective in response to prompts on wellbeing, flourishing, and yoga principles, from which three major emergent themes, nine major emergent subthemes, and 13 major categories arose (See Figure 1 for full details). Participants described feelings and multiple dimensions of wellbeing and flourishing as well as perceptions of and considerations for implementing the FLEX program (Figure 2). See Supplementary file 2 for full codebook with all MU.

No minor subthemes emerged under the theme *whole person health*. Under subtheme *dimensions of wellbeing,* four minor categories emerged including *social wellbeing* (2 MU, 2 focus groups), *nutritional wellbeing* (2 MU, 2 focus groups), *taking care of oneself* (1 MU, 1 focus group), and *enjoying hobbies* (1 MU, 1 focus group). Under subtheme *feelings of flourishing,* 10 minor categories emerged including *feeling energy or ease in daily life* (2 MU, 2 focus groups), *feeling completely balanced* (2 MU, 1 focus group), *feelings of overcoming difficulties in life* (2 MU, 1 focus group), and other categories such as *feeling creative, feeling mentally spacious, feeling that there is time for family, feeling well from nourishing foods, feelings of productivity or functioning, still flourishing even if working on bettering different areas of flourishing,* and *reciprocally radiating complete balance in to others* (each 1 MU). Under subtheme *holistic wellbeing,* two minor categories emerged including *Flourishing Index domains as a model for flourishing* (4 MU, 2 focus groups) and *ways of being* (3 MU, 2 focus groups). Under subtheme *feelings of wellbeing,* four minor categories emerged including *feeling healthy* (2 MU, 2 focus groups), *feeling comfortable with oneself* (2 MU, 1 focus group), *feeling happy* (1 MU), and *feeling energy and vitality* (1 MU). Under subtheme *key domain of flourishing,* four while minor categories emerged including *financial and material stability* (3 MU, 2 focus groups), *close social relationships* (3 MU, 2 focus groups), *happiness and life satisfaction* (2 MU, 2 focus groups), and *mental and physical health* (2 MU, 2 focus groups).

No minor subthemes emerged under the theme *wellbeing program perceptions.* Under *perceptions about yoga principles*, five minor categories emerged including *pausing or breathwork perceived as important* (4 MU, 2 focus groups), *yoga principles as part of toolkit toward mutual flourishing or Extension programming for diverse contexts* (4 MU, 1 focus group), *movement important for feeling well* (3 MU, 2 focus groups), *yoga practices perceived as beneficial for home setting, not just work setting* (2 MU, 2 focus groups), and *yoga practice perceived as accessible* (1 MU). Remaining major emergent subthemes only included minor emergent categories. Under subtheme *suggestions for FLEX program*, minor emergent categories included *reminders to do yoga practices/integration of yoga practices into work schedule* (4 MU, 1 focus group), *inclusion of play or prizes into programming* (2 MU, 2 focus groups), *inclusion of specific strategies for integrating FLEX practices* (2 MU, 2 focus groups), and other categories such as *inclusion of setting boundaries, hybrid asynchronous lectures with synchronous activity*, *inclusion of short, accessible videos,* and *partnering with community resources* (each 1 MU). Under subtheme *FLEX program is desirable*, minor emergent categories included *need for employee wellness program* (3 MU, 2 focus groups) and *desire to receive training to support implementation of personal flourishing practices* (3 MU, 1 focus group).

Under *considerations for implementing wellbeing program*, three minor subthemes emerged including *interpersonal barriers to participating in yoga or wellbeing practices* (4 MU, 2 focus groups), *individual barriers to participating in yoga or wellbeing practices* (2 MU, 2 focus groups), and *system-level support for participating in yoga practices* (2 MU, 1 focus group). Under major emergent subtheme *system barriers to participating in yoga or wellbeing practices*, two minor categories emerged including *safe and accessible spaces in which to practice movement* (2 MU, 2 focus groups) and *yoga practices are poorly understood* (1 MU). Minor emergent categories under subtheme *interpersonal barriers to participating in yoga or wellbeing practices* include *perception of generational influence on work culture* (3 MU, 2 focus groups) and *false perception that agents have it altogether or are already balanced* (1 MU). One minor category emerged under subtheme *individual barriers to participating in yoga or wellbeing practices* as *resistant views to pausing or taking a break for self-care* (2 MU, 2 focus groups). One minor category also emerged under subtheme *system-level support for participating in yoga or wellbeing practices* as *office culture of taking breaks for movement* (2 MU, 1 focus group).

#### Program materials input

**Figure 3:**
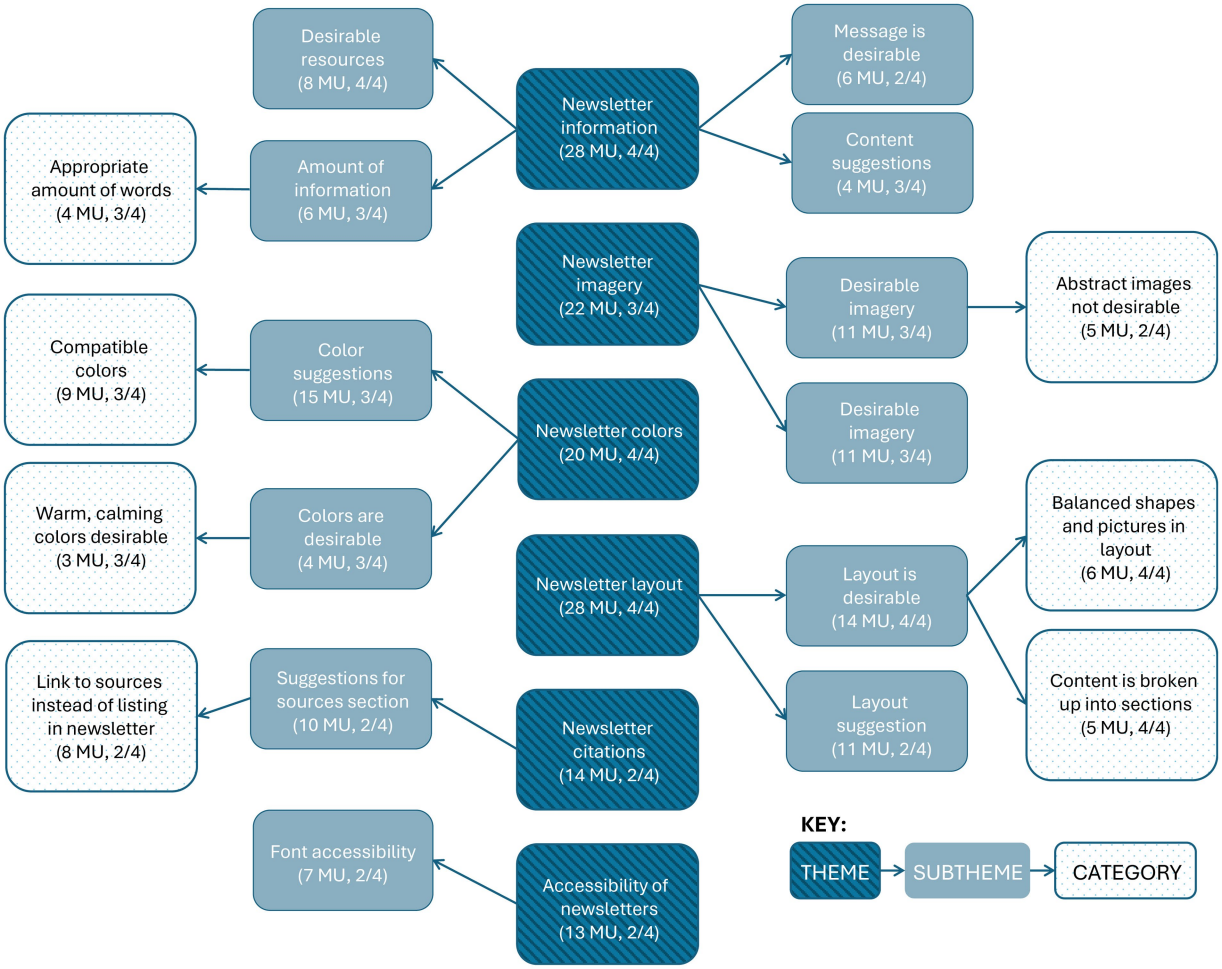
Major emergent themes, subthemes, and categories related to input on program materials. MU, meaning units.

**Figure 4:**
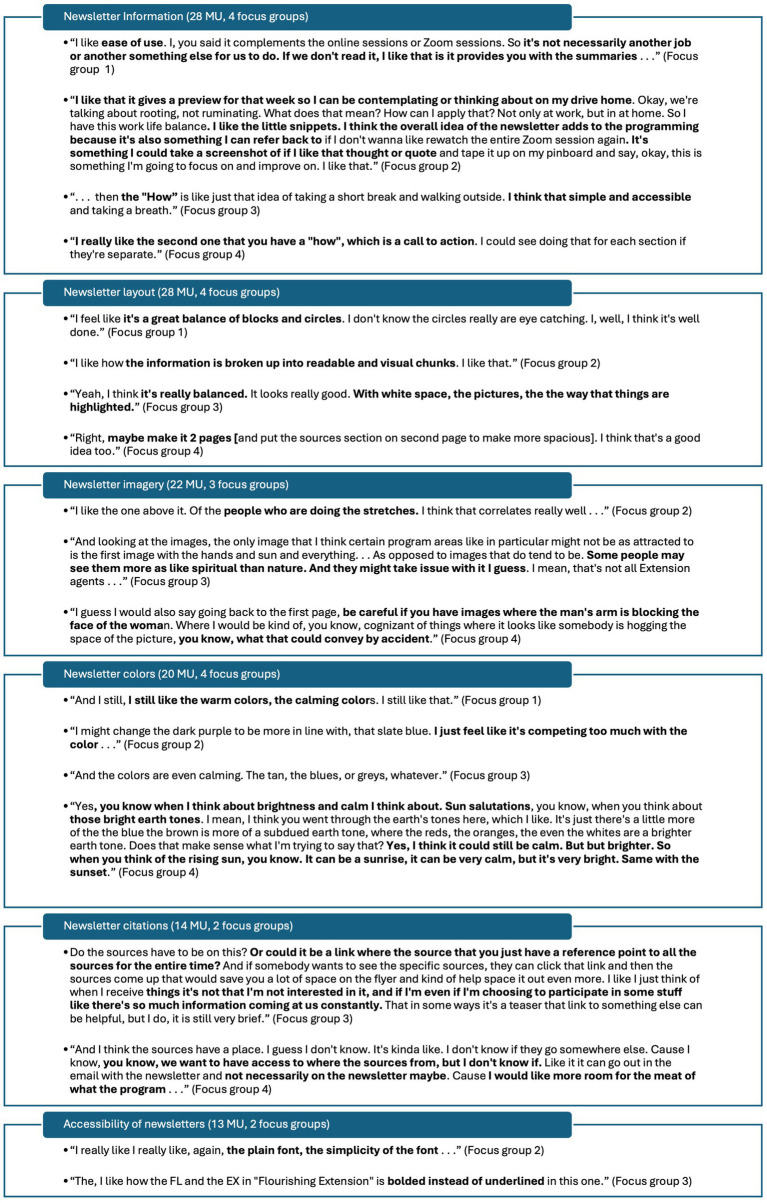
Exemplar meaning units related to input on program materials.

During the think alouds for the program materials (i.e., weekly newsletters), focus group participants provided input for future updates, from which six major emergent themes, 12 major emergent subthemes, and seven major categories arose (See Figure 3 for full details). Participants generally noted appreciation for newsletter information, layout, and colors while also providing suggestions for newsletter imagery, citations, and accessibility (Figure 4). See Supplementary file 2 for full codebook with all MU.

Under the theme *newsletter information*, one minor subtheme emerged as *Cooperative Extension content* (4 MU, 2 focus groups). Under major subtheme *amount of information*, one minor category emerged as *too many words* (2 MU, 2 focus groups). Under major subtheme *desirable resources,* minor emergent categories included *information on simple, accessible practices is desirable* (4 MU, 2 focus groups), *link or QR code to more resources is desirable* (2 MU, 2 focus groups), and *newsletters as not an addition but a takeaway to complement weekly sessions are desirable* (2 MU, 2 focus groups). Under major emergent subtheme *message is desirable,* minor emergent categories include *flourishing themes and yoga concepts in general* (3 MU, 1 focus group), *“rooting, not ruminating”* (2 MU, 2 focus groups), and *“subtle anatomy”* (1 MU). Under subtheme *content suggestions*, minor emergent categories include *removal of yoga chakras content* (2 MU, 1 focus group), *inclusion of weekly session reminder* (1 MU), and *clarity that newsletter is an educational resource* (1 MU). Under subtheme *Cooperative Extension content*, minor emergent categories include *Virginia Cooperative Extension no-discrimination statement comments* (3 MU, 2 focus groups) and *logos perceived as important* (1 MU).

Under major theme *newsletter layout*, one minor subtheme emerged as *font layout* (3 MU, 2 focus groups). Under major emergent subtheme *layout is desirable*, minor emergent categories include *one page is desirable* (2 MU, 1 focus group) and *layout is generally desirable* (1 MU). Under major emergent subtheme *layout suggestions*, two minor categories emerged as *make circular images the same size* (2 MU, 1 focus group) and *make space for information to be more spread out* (1 MU). Under major emergent subtheme *font layout*, minor emergent categories included *justify and format font to create uniform paragraph blocks, curve font headings around images, make bolding of in-text key words consistent* (each 1 MU).

No minor subthemes emerged under major theme *newsletter imagery*. Only minor categories emerged under major emergent subtheme *desirable* imagery, including *imagery of people doing yoga is desirable* (4 MU, 2 focus groups), *nature imagery is desirable* (3 MU, 2 focus groups), *inclusive is imagery desirable* (2 MU, 1 focus group), *imagery of people in groups is desirable* (1 MU), and *specific image of hands with sun* (1 MU). Under major emergent subtheme *undesirable imagery*, minor emergent categories included *specific imagery of hands with sun not desirable* (2 MU, 2 focus groups), *specific imagery of feet not desirable* (2 MU, 2 focus groups), *cartoon graphics not desirable* (1 MU), and *fewer images as an option* (1 MU). No minor subthemes emerged under major theme *newsletter colors*. Under major emergent subtheme *color suggestions*, one minor category emerged as *brighter colors more desirable* (6 MU, 1 focus group). Under major emergent subtheme *colors are desirable*, one minor category emerged as *colors are generally desirable* (1 MU).

Under major emergent theme *newsletter citations*, two minor subthemes emerged as *in-text citations suggestions* (3 MU, 2 focus groups) and *Cooperative Extensions as audience* (1 MU). Under major emergent subtheme *suggestions for sources section*, one minor category emerged as *link a list of sources all in one place for all newsletters each week* (2 MU, 2 focus groups). Under subtheme minor emergent subtheme *in-text citations suggestions*, two minor categories emerged as *use superscript instead of parentheses* (2 MU, 1 focus group) and *do not bold in-text citations* (1 MU). The one minor emergent category under minor emergent subtheme *Cooperative Extension as audience* is *scholarly, research-based information okay for agents as audience* (1 MU). Additionally, under major emergent theme *accessibility of newsletters,* two minor subthemes emerged as *considerations for emailing newsletters* (3 MU, 2 focus groups) and *colors contrast* (2 MU, 1 focus group). Under major emergent subtheme *font accessibility,* minor emergent categories include *bold instead of underline FL EX in Flourishing in Extension* (3 MU, 2 focus groups), *accessible font with font hierarchy is desirable* (3 MU, 1 focus group), and *justify font left instead of center* (1 MU). Under minor emergent subtheme *considerations for emailing newsletters,* minor emergent categories include *too many images will make pdfs too large for some computers*, *emails will block images in email body* and *add link in addition to QR code* (each 1 MU). Under *colors contrast*, one minor category emerged as *use blues instead of brown* (1 MU).

#### Recruitment materials input and adaptations

**Figure 5:**
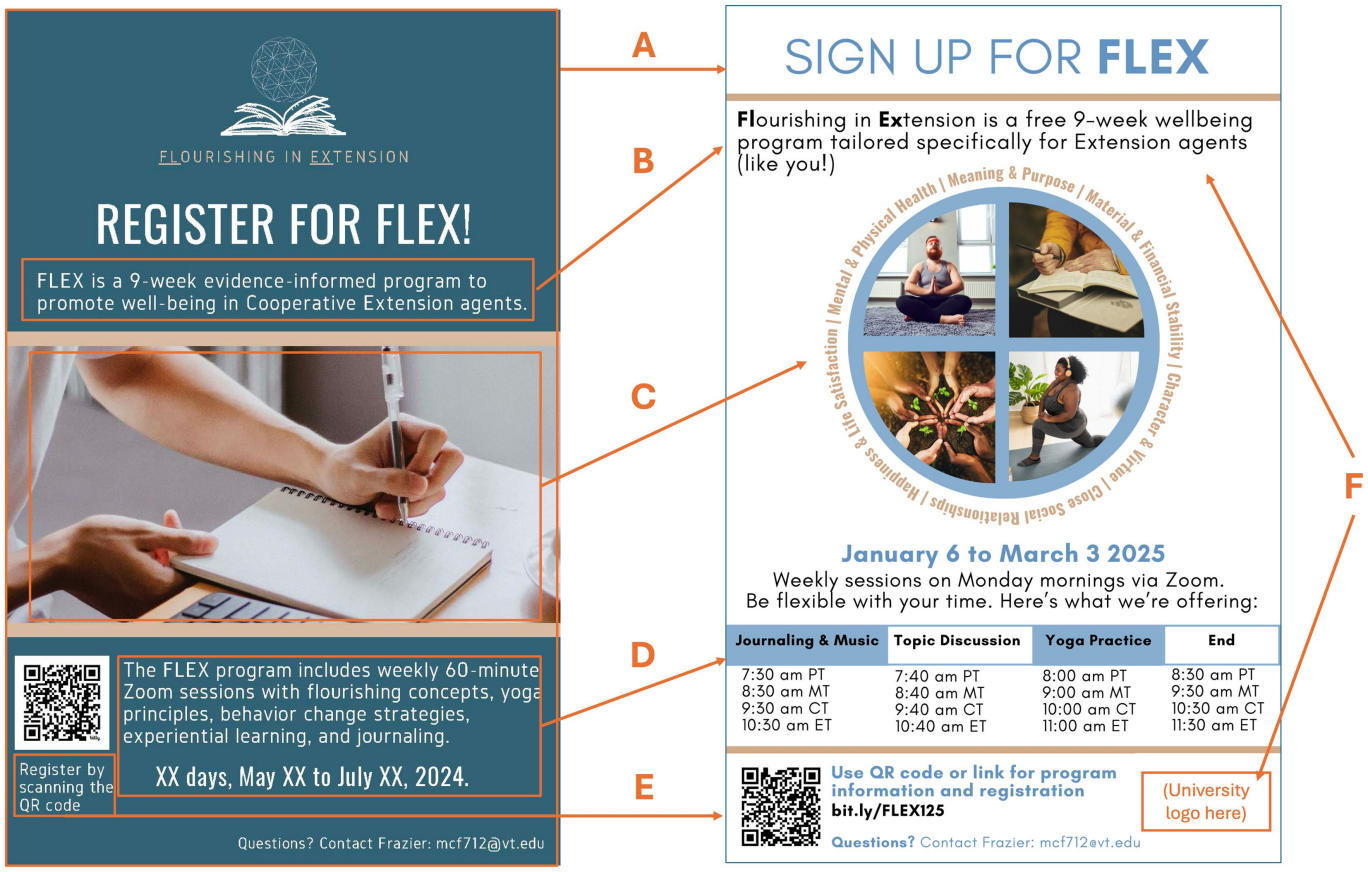
Comparison of intervention newsletter before and after data-driven adaptations. **(A)** Kept desirable components (e.g., kept QR code); **(B)** improved font accessibility (e.g., dark font against light background); **(C)** created figure to illustrate program components and desired imagery (e.g., included multiple, inclusive images of yoga practices and journaling); **(D)** added graphic of time frames with corresponding activities for weekly session; **(E)** added clickable link; **(F)** changed language and added university logo to provide better clarity and credibility.

During the think alouds for the recruitment flyer, focus group participants provided input for future updates (Figure 5; Table 2). Input included (A) desirable flyer aspects including calm colors, QR code, simple and straightforward design, program name, and accessible font (18 MU, 4 focus groups); (B) suggestions to improve font accessibility including using dark font on a light background, bolding instead of underlining, and making the text larger and more clear (11 MU, 4 focus groups); (C) suggestions to make the central figure more reflective of the FLEX program by including multiple images to show the different aspects of FLEX such as yoga, breath, journaling, flourishing domains, etc., as well as make imagery inclusive with different sized bodies and different skin colors (23 MU, 4 focus groups); (D) suggestions to add information about “drop in” time frames for weekly session activities (14 MU, 3 focus groups); (E) suggestions on providing clarity such as specifying that the program is free, which university the program is from, and that sessions are via Zoom (8 MU, 3 focus groups); and (F) suggestions to use a short link in addition to the QR code (5 MU, 2 focus groups).

**Table 2:** Input-driven adaptations to recruitment materials with key of adaptation types.

Key	MU	n of FGs	Exemplar meaning unit (MU)	Researcher notes	Updates
A	18	4	**I love the color…**it’s **very calming**. It’s not too in your face (FG 2)	Calm colors desirable	Keep blue and tan color scheme
			Okay. And the **QR code**. That’s **fantastic**. (FG 1)	QR code desirable	Keep QR code
			I like that it’s **simple** and **straight to the point**. (FG 3)	Simple, straightforward design desirable	Keep simple and straightforward
			[Participant] said **she liked the word FLEX**, and she put a little **muscle emoji** [in the Zoom chat]. **Flourishing in Extension is FLEX, haha**. (FG 4)	Program name is desirable	Keep ‘FLEX’ as program name
			**I like that it’s geared toward Cooperative Extension agents**... that it makes you feel like the treatment is **tailored to some things that are unique to you.** (FG 1)	Tailored for Cooperative Extension agents content desirable	Keep “tailored for Cooperative Extension agents” content
B	11	4	And then I just have a little **trouble reading the font** on that light colored font. It’s kinda hard. (FG 4)	Accessibility: Font colors - better to have dark font on light background than vice versa	Made font blue against white background
			Um, I, in the name, “Flourishing in Extension,” **I would not underline the FL in the EX, but I would put it in bold.** The **underlining takes away from the letters** that are actually there. (FG 2)	Accessibility: Bold instead of underline F-L and E-X in “Flourishing in Extension”	Bolded F-L and E-X in “**Fl**ourishing in **Ex**tension”
			The “Flourishing in Extension,” **I agree with the comment to make that bigger.** (FG 3)	Accessibility: Make “Flourishing in Extension” font larger	Made font larger, clearer
C	23	4	I thought about the imagery too. **And I know that everybody is different on imagery**. Some people do not want it to be too much. Some people like to see more. **But I thought about maybe, having 4 blocks of different aspects of some of the domains that you told us.** (FG 1)	Have multiple images to show the different aspects of FLEX	Created figure of multiple images (i.e., inclusive yoga, journaling, and “flourishing” plants) with names of flourishing domains
			I think I want to see more of the the things on the bottom reflective in the picture, **not just journaling, but maybe the yoga**. And and like **diversity** in the pictures. **Whether it is the different sized bodies, the different color skins, just seeing that is this is for everybody**. **Because sometimes in the marketing I sometimes feel left out and just because of what’s reflected on the flyers**. And a lot of times in some spaces that I’m in. **Yoga is definitely looked at not always a safe place for Black people**. So, especially, so that kind of stood out to me that, you know, **if we are going to say yoga, I want to make sure it’s reflective of all people are welcome to do this.** (FG 4)	Inclusive imagery is important. Have multiple images to show the different aspects of FLEX	
			I agree the trickiness of choosing images because I have like a wellness lunch and learn series that I was trying to get pictures for and **it was just like nothing felt right especially with like picking pictures for yoga because** there’s very much like, **especially if you are using Canva, there’s like one type of person that’s like portrayed in those like free images**. And you are kind of like well I, you know, **I do not want to turn off anybody who maybe does not look like this person**. So, I definitely agree it can be kind of difficult... So, I think **maybe like a flower or something blossoming might be like very like Extension coded just like with agriculture agents -** different things like that like forage. so I think you know, maybe a picture like that, **cause we talked a lot about how flourishing reminds us of growth**... So that might be just something to consider. (FG 3)	Inclusive imagery is important. Consider plant and nature imagery.	
			And **the image is pretty clean,** nice, but for me it’s like too pure. **Too clean. Too kind of bland.** (FG 2)	Too bland - add vibrancy by using multiple images.	
D	14	3	**Can you explain what a typical 60 min session would look like?** That might help us better pinpoint things that would, **help your audience relate to the poster.** (FG 2)	Show schedule of activities for weekly sessions (60 min total, but first 10 min is XYZ, last 30 min is ABC, etc.)	Added graphic of time frames with corresponding activities for weekly session
			I think what **what pops out to me as far as makes me go, is, ugh, 60 min… And it’s hard to find that time**… they may seem “yeah need to I really need to get better about being active,” but when it comes down to, they do not want to give up any of their little bit of time... **So, getting, drawing them in and getting them to do a 60 min Zoom every week. I think it’s gonna be difficult.** (FG 1)	Show timeframes for weekly sessions	
			**My other concern would be not having the time of when the Zoom sessions are.** I do not know if that means you can take it whenever you want… (FG 4)	Need clarity on start time and whether sessions are live or self-paced	
E	4	1	**I would add a link** because some people do not use their phones necessarily, but a clickable link. [FG 2]	Add clickable link	Added clickable link
F	8	3	I live in an area where **a lot of people are worried about finances**. And so, knowing if the class is free or if there is a cost would be helpful and **even as Extension educators as we are managing our own funding.** (FG 2)	Show costs (free)	Specified free program
			... **I also think that when you say a university is providing evidence informed programs that that, lends credibility to it.** [FG 4]	Need to add university logo	Added university logo
			... **Behavior change strategies.** I think somebody else kinda hit on this like. The reaction could be like, why do, why do we need this? **And I do not know it can be off-putting.** [FG 3]	Omit “behavior change strategies” (consider “strategies for personal growth”)	Removed “behavior change strategies” content

MU, meaning unit count. FG, focus group.

### Follow-up survey findings

**Figure 6:**
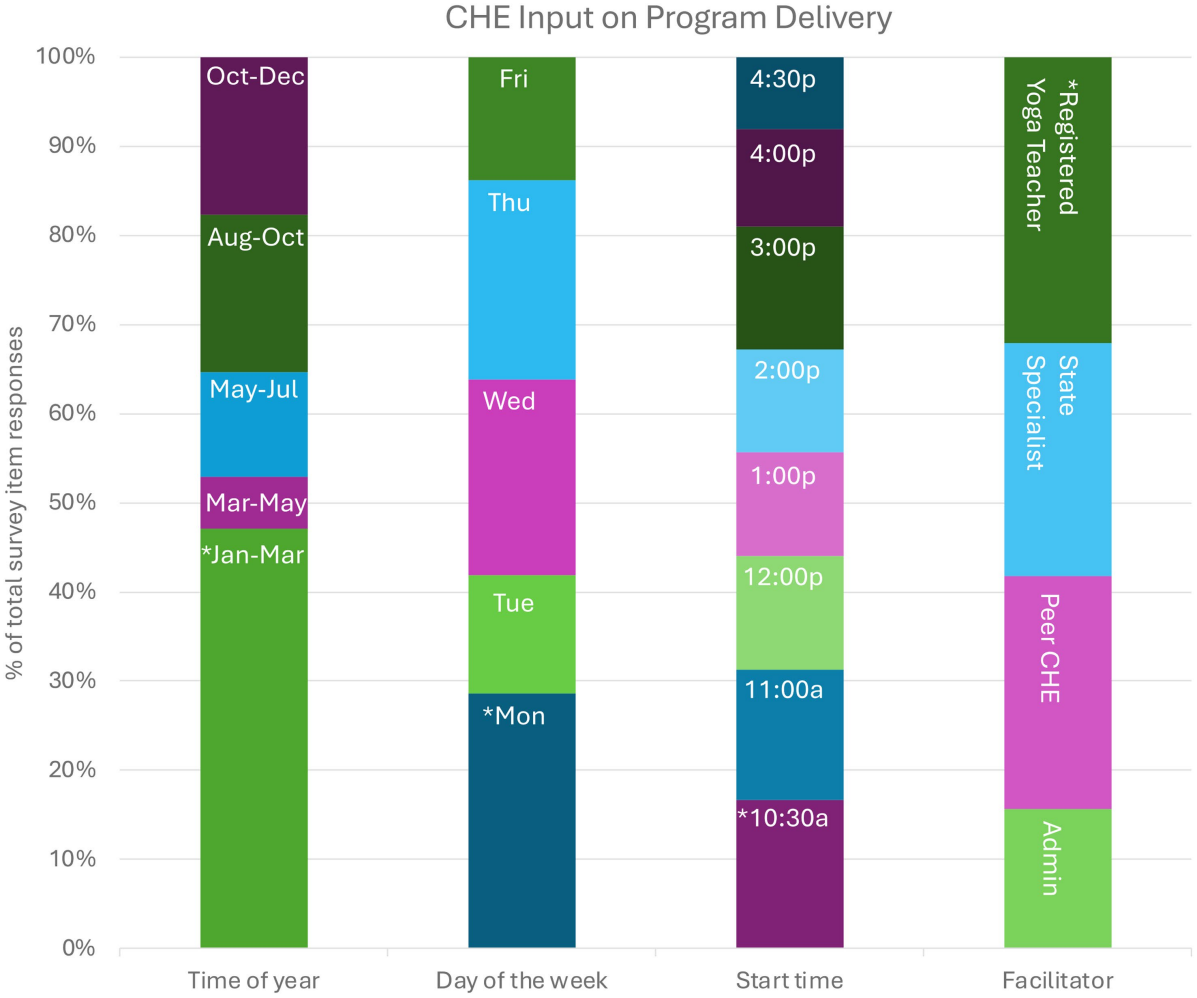
Follow-up survey findings based on input for program delivery factors. *Asterisk indicates greatest proportion of participant selection. Months and weekdays are abbreviated using the first three letters of names. Start times were controlled for different time zones and are represented here using Eastern Time. CHE, community health educators. ‘p’ indicates PM. ‘a’ indicates AM.

Participants who completed the follow-up survey represented four time zones: 1 in Pacific Time (6%), 2 in Mountain Time (12%), 3 in Central Time (18%), and 11 in Eastern Time (65%). For intervention delivery, CHE overall preferred program weekly session to be during January–March on Monday mornings and facilitated by a registered yoga teacher (Figure 6). In response to the program facilitator prompt, two participants selected the “other” open-ended option, writing that a facilitator could be “anyone certified in workplace wellness, yoga, stress management, etc.” and that it “depends on their level of personal practice and experience.”

**Figure 7:**
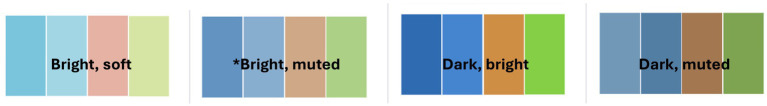
Color palette options provided in follow-up survey. *Asterisk indicates greatest proportion of participant selection.

In response to prompts on program materials, participants ranked color palettes and options for weekly sessions order-of-events according to preference. In one focus group, a participant used the chat feature to upload a photo that demonstrated their preferred hues and colors. Color palettes based on this photo were then created and shared for direct feedback in the remaining focus groups. Of the color palettes, participants ranked the ‘bright, muted’ highest (Figure 7). Of the options for weekly sessions order-of-events, participants ranked highest the following order-of-events: 10-min journal prompt, 20-min education with discussion, and 35-min guided yoga practice.

In response to prompts on interest in participating in a pilot program of FLEX, 15 survey participants selected ‘yes’ (88%), one survey participant selected ‘no’, and one survey participant selected the ‘other’ option (6%), noting that: “I am [interested] but I’m not sure if my schedule will allow it.” When those interested were asked whether they preferred to participate in the pilot program in June 2024 versus September 2024, 8 participants selected September (53%) and 4 selected ‘other’ (27%), noting that summer and fall times are busy months and that November–February would be best. Two participants (12%) provided additional information in response to an optional prompt at the end of the survey, with one participant corroborating the previous statements that summer and fall times were busy times. The other participant, stated:

I really like the FLEX idea and would really enjoy participating. I did find over the last number of weeks that there was ALWAYS something that came up that I couldn't avoid. I would imagine that is the case for many agents. **I think there are probably solutions for this, but it may take a lot of patience while figuring it out.**

### Data-informed program adaptations

**Figure 8:**
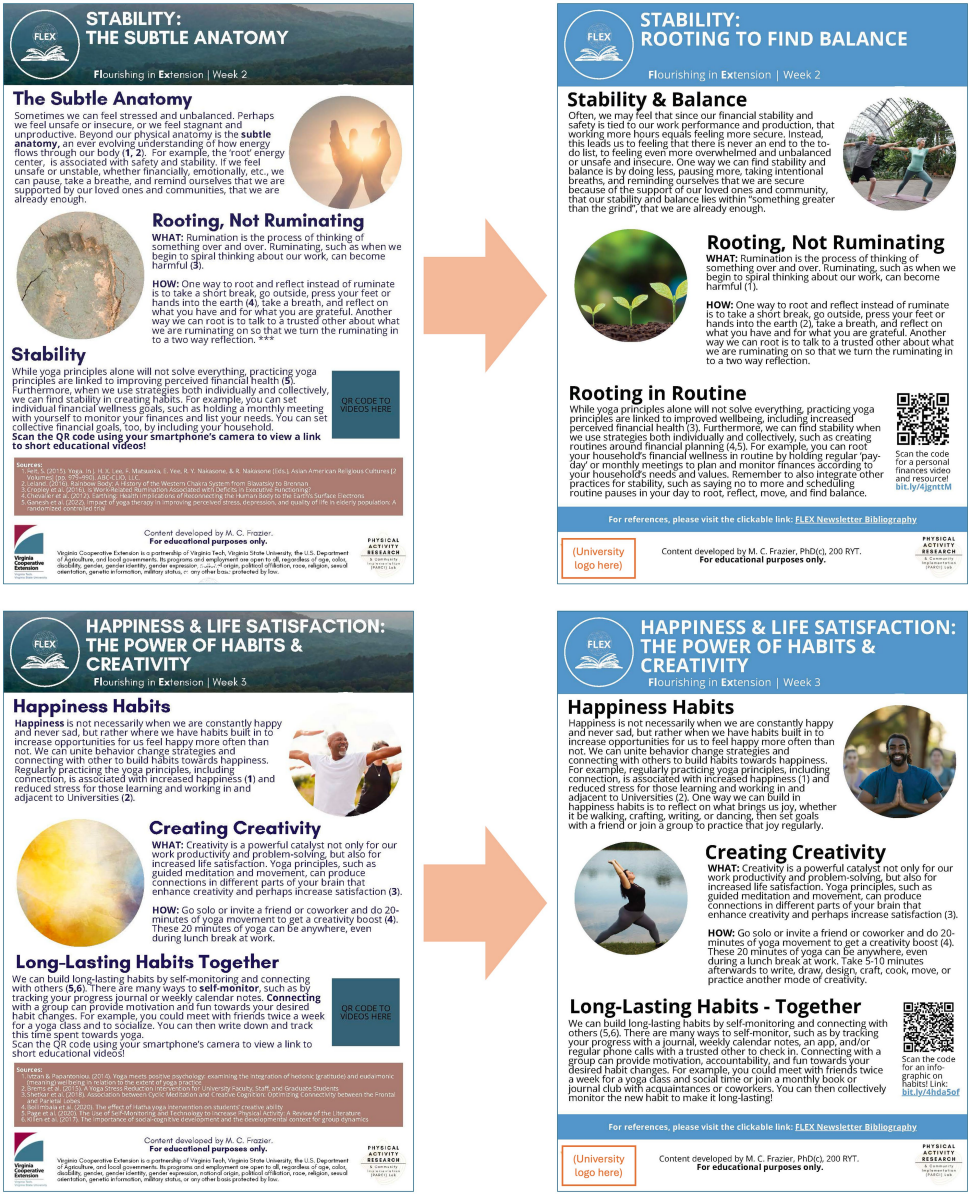
Before and after comparisons of week 2 and week 3 newsletters based on input.

Mixed methods data from IRPP members and national CHE focus group sessions with a follow-up survey informed several adaptations (Table 3). First, the national launch of the 9-week pilot FLEX program was rescheduled for January 2025 with weekly sessions on Monday mornings to align with CHE input on scheduling. Additionally, the recruitment flyer was updated based on data from focus group think-alouds (Figure 5). FLEX program materials were also updated based on data from both focus groups and follow-up survey findings (Figure 8).

**Table 3:** Pre-implementation process for capturing contextual, co-created intervention adaptations, mapped to the ADAPT framework (71, 72).

ADAPT step	Co-creation channel	Key activities
Step 1 Assess rationale for intervention and consider intervention-context of existing interventions.	Participatory approach* with CHE of one state Cooperative Extensions *e.g., IRPP (52), sometimes called participatory action research (86, 87)	Listened to needs and surveyed perceptions of CHE members of IRPP Reviewed prior employee wellness programs for CHE (42, 43) Discussed and co-created initial adaptions program functions and forms with IRPP members
Step 2 Plan for and undertake adaptations.	**Focus group and a follow-up survey** with wider sample of CHE from multiple state Cooperative Extensions	Conducted focus groups with CHE from multiple states (*N* = 21, *n* = 4 to 6 per session, 9 states represented) Conducted a follow-up survey with focus group participants (*n* = 18) Co-created adaptations to program content and delivery based on focus group and follow-up survey data
Step 3 Plan for and undertake piloting and evaluation.	Next step from this pre-implementation study	Next step from this pre-implementation study
Step 4 Implement and maintain adapted intervention at scale.	Not applicable for this pre-implementation study	Not applicable for this pre-implementation study

IRPP, integrated research-practice partnership. CHE, community health educator.

## Discussion

In this sequential mixed-methods pre-implementation study of an employee wellness behavioral intervention for CHE of the federal Cooperative Extension System, we provide three key findings. First, we describe CHE perceptions on wellbeing, flourishing, and yoga principles to inform the future development and implementation of employee wellness intervention core functions. Second, we demonstrate specific input-driven adaptations to preexisting wellness program core functions and forms for Cooperative Extension, resulting in the FLEX program. Lastly, we share insights for the process of contextually tailoring employee wellness interventions for populations at risk for or experiencing burnout such as CHE.

With the exception of previous phases of FLEX (42), prior peer-reviewed research on worksite wellbeing programs for CHE of Cooperative Extension, to the best of the authors’ knowledge, do not exist. However, a systematic review of virtual worksite mental health interventions for knowledge sector employees (i.e., business, communication, education, finance, and information-related research) demonstrated small positive effects for improved psychological wellbeing (e.g., anxiety, depression, and stress) and work effectiveness (i.e., productivity and engagement) (18). Although use of participatory methods and facilitation costs were not analyzed in the reviewed interventions (mean duration 7.6 weeks), over half were reported to be self-paced while the remaining were mostly guided by a therapist or coach (18). Notably, more than half of the interventions used a cognitive behavioral therapy approach which demonstrated small effects for psychological wellbeing compared to the medium effects of psychological approaches of other studies, one of which used positive psychology (e.g., happiness) (66). One reason for these small effects of worksite interventions is that the functions of these intervention may not align with the nuances of what employees need and value most for flourishing in the workplace (26). One systematic review on job flourishing research specifies that more dynamic functions of worksite interventions may serve beyond short-term organizational (e.g., burnout prevention) toward more humanistic goals, such as community embeddedness and health (67). In fact, in our focus group data, CHE of Cooperative Extension described a *whole person health* of which holistic wellbeing encompasses *a multi-dimensional system*, including the *mental*, *physical*, *emotional*, *spiritual*, and *social* dimensions. CHE descriptions of whole person health and wellbeing align with the biopsychosocial-spiritual model (68) and flourishing index (20, 27). CHE further described *key domains of flourishing*, particularly *meaning and purpose*, as well as *feelings of flourishing*, particularly *feelings of thriving* and *of exceeding one’s potential as a continuum*. These data demonstrate target positive responses (i.e., internal affect) for future intervention behavioral change mechanisms (69). Additionally, CHE perceptions of yoga principles were overall positive and demonstrated the desire for *yoga principles as a part of a toolkit for diverse audiences to use toward their personal flourishing*. As the health behaviors of CHE can influence the health of the people they serve (49, 50), future CHE employee wellness interventions may consider how the provision of core functions based in a biopsychosocial-spiritual wellness approach for CHE would build their confidence and competence to facilitate the same practices toward flourishing for the communities they serve (e.g., offering journal prompts or breathwork in workplaces).

To align with input from triangulated participatory, qualitative, and quantitative findings (47), we made needs-based, data-driven adaptations to the core functions and forms of the FLEX program, particularly regarding the schedule of delivery and program materials. While we initially prepared to deliver the program in the summer or fall of 2024, we adapted our approach based on overwhelming data that the winter months would be best for CHE busy schedules. We instead scheduled delivery of the program for January 2025 after winter holidays based on CHE data. Additionally, we adapted program materials to align with CHE input based on major emergent themes. For example, several participants noted the need to see a timetable or graphic on the recruitment flyer so that it was clear what FLEX weekly sessions entailed to inform CHE decisions on whether to sign up. Two participants from two different focus groups also noted that specifying whether the FLEX program was free provided important clarity for recruitment. For program materials (i.e., emailed newsletters), participants noted sufficient information but suggested using more clearly relevant imagery instead of imagery perceived as abstract or unclear. Additionally, we made adaptations based on minor emergent themes of low effort and possible high impact, such as adding clickable links to recruitment and program materials and making shapes and font consistent. We also incorporated feedback on content and accessibility of program materials. As examples, CHE provided key insights into use of imagery, such as using multiple images to demonstrate inclusivity and provide clarity on the multiple program components, as well as suggested improvements for accessibility, including color contrast and using accessible font. Furthermore, CHE provided valuable insights into implementing a wellbeing program by naming barriers at multiple levels, most notably *system barriers to participating in yoga practices*. As CHE described a *culture of busyness* as a barrier to participating in personal flourishing practices, future CHE employee wellness intervention adaptations and development may consider tailoring intervention forms for hierarchal key decision-makers in CHE systems, such as regional administrators of Cooperative Extension Systems. Overall, these co-created adaptations provided rich insights for tailoring the intervention and also served as a reminder of the patience required in facilitating participatory work (47). These findings further corroborate prior work on methods for understanding end-user needs and contextual factors and intervention core functions and forms to ultimately improve intervention-context fit (45, 46).

This process of capturing contextual adaptations can be replicated by others to improve intervention acceptability and sustainability (70–72). Notably, this process can be applied at the pre-implementation phase as well as iteratively after intervention delivery. Crucial to this adaptation and tailoring process is co-creation: the participatory, collaborative, and iterative approach with intervention decision-makers and end-users to design and problem-solve at all levels of intervention research (73, 74). Based on mixed methods for co-creation (75), this study outlines collaborating with participatory partners to adapt existing intervention core components and functions and then using focus groups with user-centered think alouds and a follow-up survey with a wider sample to collect input on program forms (i.e., characteristics, delivery, and materials) to inform adaptations for improved reach and acceptability. This qualitative process may be bolstered by using models to guide pre-implementation data collection including premortem brainwriting (76); the Exploration, Preparation, Implementation, and Sustainment model (77, 78); and the Practical, Robust Implementation and Sustainability Model (79, 80). While co-creation is a valuable practice for all stages of intervention development and testing, we found that garnering end-user input at the pre-implementation stage may not only improve fit of program characteristics, delivery, and materials but also reveal crucial nuance to program scheduling and delivery, especially for burnout populations already experiencing high burden and time scarcity (5, 81, 82). Beginning and adapting wellbeing interventions with the end-user in mind (83, 84) is thus integral for contextually competent dissemination and implementation. This approach overall aims for interventions to reach and resonate with those who would benefit from them most, like how one focus group participant shared: “[It] makes you feel like that the program is designed by people that have you in mind.”

### Strengths and limitations

One prominent strength of this study is the use of rigorous participatory and mixed methods to co-create an adapted intervention at the pre-implementation context. Another strength of this study is the focus of an understudied employee population with inclusion of community health educators from multiples states. An additional strength of this study is the use of an existing IRPP of over 10 years (51). For investigators that do not have an already established partnership, many methods from engagement research, community based participatory research, and dissemination and implementation science (47, 48) could be applied to begin building partnerships, such as identifying key decision-makers and developing goals and role clarity. Additionally, we took a novel approach to major and minor emergent qualitative analysis by allowing quantitative divides in the data to determine thresholds. This study also contains limitations. First, the study lacks a guiding implementation model or framework for qualitative analysis. Second, while the knowledge of the coding researchers about CHE, flourishing, and yoga may serve as a strength, this positionality may also unconsciously contribute to bias. However, both coding researchers completed training in inductive qualitative analysis and were instructed to stay as close to the source material as possible to mitigate possible bias. Additionally, our findings may not be generalizable to wider populations as our study samples are small and predominantly female and White. However, these sample characteristics may be representative of the CHE population according to other studies (85) although there is a lack of national statistics to verify this. Furthermore, sample recruitment may be subject to potential selection bias. Lastly, our study does not include pilot testing validation of the focus group prompts.

### Future directions

Future methods include assessing if adapted materials meet preferences and expectations of participants as well as testing potential reach and acceptability of these materials with prospective participants who did not contribute to this formative work. Future directions of this work also include launching a mixed methods feasibility pilot study of the adapted, co-created FLEX program to test the acceptability of program characteristics and implementation.

## Conclusion

This mixed methods pre-implementation study captured often overlooked contextual factors important for intervention feasibility, acceptability, and implementation. We learned that collecting input and feedback from target burnout populations may serve as an important implementation strategy for improving future recruitment and retention. By collecting target population-specific data, especially from underrepresented, burnout, and other hard to reach and retain populations, valuable input can inform co-creation and adaptations of behavioral health intervention characteristics and delivery and ultimately increase accessibility, representativeness, and efficacy.

## Data Availability

The raw data supporting the conclusions of this article will be made available by the authors, without undue reservation.
