# Supplementary material for: Patience is a virtue: lessons from a participatory approach to contextually tailor and co-create an employee wellness intervention for community health educators
Source: Front Public Health. 2025 Sep 19;13:1634264. doi: 10.3389/fpubh.2025.1634264 (PMC12491061; doi:10.3389/fpubh.2025.1634264)
Supplement: Supplementary file 1 [file Table_1.docx]

**Supplementary Table S1**: Initial week-by-week guide of the Flourishing in Extension program

| **Week** | **Principles / Topics** | **Behavior change technique (BCT)** |
| --- | --- | --- |
| 1 | Yoga: The What, Why, and How | Physical activity prescription Provide education |
| 2 | Stability: The Subtle Anatomy | Physical activity prescription  Self-monitoring Motivational interviewing  Personalized messages  Provide education |
| 3 | Happiness & Life Satisfaction: The Power of Habits & Creativity | Physical activity prescription Provide education Self-monitoring  SMART goal setting Support self-efficacy |
| 4 | Meaning & Purpose: Personal Values, Goal Setting, and Purpose Mapping | Physical activity prescription Provide education  Motivational interviewing  Goal setting |
| 5 | Relationships: Yoga for Connection, Community Contexts, and Public Health | Physical activity prescription Provide education  Self-monitoring  Problem-solving barriers |
| 6 | Character & Virtue: Self-Esteem, Confidence, and Empowerment | Physical activity prescription Self-monitoring  Motivational interviewing |
| 7 | Holistic Health: Mindful Movement and Intuitive Eating | Physical activity prescription  Intuitive eating [82,83] Small changes  Self-monitoring |
| 8 | Flourishing: Rest and Stress Management for You and Your Lifestyle | Physical activity prescription Provide education  Small changes Self-monitoring Goal setting |
| 9 | Sustainability & Growth: Maintaining our Habits and Sharing with Others | Physical activity prescription  Problem-solving barriers  Self-monitoring Relapse prevention  SMART goal setting Support self-efficacy |
