# Supplementary material for: Patience is a virtue: lessons from a participatory approach to contextually tailor and co-create an employee wellness intervention for community health educators
Source: Front Public Health. 2025 Sep 19;13:1634264. doi: 10.3389/fpubh.2025.1634264 (PMC12491061; doi:10.3389/fpubh.2025.1634264)
Supplement: Supplementary file 2 [file Table_2.docx]

**Supplementary Table S2**: Full codebook for focus group qualitative data.

| **Focus Group** | **Prompt** | **Meaning Unit (MU)** | **Category** | **Subtheme** | **Theme** |
| --- | --- | --- | --- | --- | --- |
| 1 | Additional comments | Well, I just love the subject matter and I love how unique this is. I'm. And I'm incredibly curious as to what we could learn from this program, really to be honest. | Desire to receive training to support implementation of personal flourishing practices | FLEX program is desirable | Wellbeing program perceptions |
| 1 | Additional comments | I've been saying it for some time among my, we have family consumer health sciences, so we're FCS here, but in the sense I've, I'm fearful for the legacy of an agent. And our newer agents coming up or are we are? I know that I come and I have big shoes to feel when it comes to being an agent, working 60 plus hours a week, you know, research this program that, you know, get grants, hold staff, we know all of that. Like, are we having generations coming up that are going to be willing to do that? And I feel like all of the research and all this very, very much life work balance, very balancing. I, I don't know if we're. And I'm not saying this to be negative. I mean, maybe putting their foot down is a great thing. You know, 40 hours is 40 hours and that's all we're doing, you know, in a week kind of thing. And, you know, I've been told forever that that's not what this job is, right? You know you work till you get the job done. You'll your to-do list. I might have a to-do list that actually gets all crossed off maybe twice a year. That's just a magical time in the universe, that it all comes back. | Culture of busyness | System barriers to participating in yoga practices | Considerations for implementing wellbeing program |
| 1 | Additional comments | And so anyway, I'm just like. I want to help, you know, those that are coming up and, how I can better model maybe more work life balance . . . | Need for employee wellness program | FLEX program is desirable | Wellbeing program perceptions |
| 1 | Additional comments | . . . and also, You know, champion being an agent and how fantastic it is. I know we're not all weary and exhausted and burned out and. We shouldn't be. This should be really great work that we're always, always excited to do and and sometimes I'm finding among colleagues that's absolutely not the truth. |  |  |  |
| 1 | Additional comments | I was just about to say the same as you (legacy of Extension), I feel like we're not, we feel it but we're not supposed to say it. | Culture of busyness | System barriers to participating in yoga practices | Considerations for implementing wellbeing program |
| 4 | Additional comments | One of the guys is in a very rural town and he was like and it was all about you know health and insurance and accessing insurance but we also talked about health and well-being and things like that. We had prizes and so he won a like a blender, like a ninja blender, a little one and he's like this is the best benefit I've ever gotten on the job. And I was like, wow, I'm really glad you liked it. | Include play/prizes into programming | Suggestions for FLEX program | Wellbeing program perceptions |
| 4 | Additional comments | So anyhow, I think they're impactful programs, and I hope it goes really well for you because I think Extension especially when they're off campus don't have access to. Wellness programs all the time especially in rural areas. I'm in an urban area, but I thought you know they used to come out and do employee health fairs like in different areas and then they just stopped doing it and this was before COVID and so I thought well we don't have any type of employee wellness. So. For extension in place especially off campus so I think it's awesome. | Need for employee wellness program | FLEX program is desirable | Wellbeing program perceptions |
| 4 | Additional comments | I'm also I think is awesome too for that same reason. And I guess I had a question about like. I guess the implementation. Because I know here where I'm at in North Carolina. All the breath work and all the. Those types of classes are offered. You know, in the community already. And I guess how could we incorporate the community? Into like to kind of create a collaboration with community and Extension. So it kinda doesn't feel like, oh, we're coming in and we're doing this. But it's been been, you know, it's been happening, I guess, cause we have such a big community and I guess our community. People here a lot of times we can't wait on sometimes the research to come with, cause like you said, it's going to take while. So I guess is there any room for bringing in the community is already doing this type work into this project? | Partnering with community resources | Suggestions for FLEX program | Wellbeing program perceptions |
| 1 | Flourishing | To me it's all those things you mentioned (6 domain of Flourishing Index) and it's just your overall being. | Flourishing Index domains as a model for flourishing | Holistic wellbeing | Whole person health |
| 1 | Flourishing | Is just your purpose, your sense of who you are the well-being is just your purpose, your sense of who you are, the wellness for mental health of. Who you are and what do you bring to the table. | Meaning and purpose | Key domain of flourishing | Whole person health |
| 1 | Flourishing | I always thought that like my goal in life was just to be happy. | Happiness and life satisfaction | Key domain of flourishing | Whole person health |
| 1 | Flourishing | You know, and be as physically well as you can be and mentally . . . | Mental and physical health | Key domain of flourishing | Whole person health |
| 1 | Flourishing | . . . not have to worry about you know that you've got bills you can't pay. | Financial and material stability | Key domain of flourishing | Whole person health |
| 1 | Flourishing | And you have time to spend with your family . . . | Feeling that there is time for family | Feelings of flourishing | Whole person health |
| 1 | Flourishing | and that your career is. . . You're being able to do something that's giving back. I recently was able to switch to a job as the FCS agent, family, consumer science. So I think I'm in a better position now than I've ever been to be able to help other people. | Meaning and purpose | Key domain of flourishing | Whole person health |
| 1 | Flourishing | I feel like, success would be in all 6 of those realms. . . | Flourishing Index domains as a model for flourishing | Holistic wellbeing | Whole person health |
| 1 | Flourishing | Or I've attained my ideal and and whichever of those 6 realms. | Feelings of thriving | Feelings of flourishing | Whole person health |
| 1 | Flourishing | Feeling like I'm where I want to be. And perhaps even. Still in control. Not in a bad way, you know, just kind of feel like I got this, you know, kind of thing. I'm in a place and a space where I can move up and down or I have the ability to. | Feeling of exceeding one's potential as a continuum | Feelings of flourishing | Whole person health |
| 1 | Flourishing | I was I was thinking along those same lines, I was thinking that flourishing for me in those areas mean that if I was thriving in all those areas to my satisfaction and if I felt like they were also in balance. And I felt overall sense of contentment because I felt like I was thriving in all those areas. | Feelings of thriving | Feelings of flourishing | Whole person health |
| 2 | Flourishing | For me, I would say am I meeting or exceeding my potential. And like, yes, we can be happy. Yes, we can meet all those things, but in my meeting, my potential like. Am I accomplishing all that I've set out to do for this day for this week? Am I worth where I thought I would be at this point in my life? That sort of a thing. | Feeling of exceeding one's potential as a continuum | Feelings of flourishing | Whole person health |
| 2 | Flourishing | Yeah, I like that, (participant name). That meeting or exceeding. Where am I at in this equation? Like on a, on a continuum. | Feeling of exceeding one's potential as a continuum | Feelings of flourishing | Whole person health |
| 2 | Flourishing | I'm thinking and then looking at the at the domains of flourishing, and then thinking how much of that I have control of. | Flourishing Index domains as a model for flourishing | Holistic wellbeing | Whole person health |
| 2 | Flourishing | I love that perspective (how much have control of). What is part of what of it is my choice. The way I look at it, the way I perceive it? My reality versus my perception. | Flourishing Index domains as a model for flourishing | Holistic wellbeing | Whole person health |
| 3 | Flourishing | I was gonna say, just looking at your definitions, getting through daily life and activities with minimal. . . I don't want to say "strife" but "easily". Not stressful. | Feeling energy / ease in daily life | Feelings of flourishing | Whole person health |
| 3 | Flourishing | Being generally happy . . . | Happiness and life satisfaction | Key domain of flourishing | Whole person health |
| 3 | Flourishing | . . . and you know, being productive and functioning. | Feelings of productivity / functioning | Feelings of flourishing | Whole person health |
| 3 | Flourishing | So yeah, I would say for me thinking of flourishing. It's like such an exciting term where you think of flourishing is getting over challenges in your life. For me, I know, you know, there's certain areas I don't necessarily flourish in and those are the ones that I'm trying to work on more . . . | Feelings of overcoming difficulties in life | Feelings of flourishing | Whole person health |
| 3 | Flourishing | . . . and when I think of areas, I think, you know, social well-being is really important. | Close social relationships | Key domain of flourishing | Whole person health |
| 3 | Flourishing | Overall wellness when it comes to like your eating, your physical activity. And then mental well-being. | Mental and physical health | Key domain of flourishing | Whole person health |
| 3 | Flourishing | So, that to me is like if you if you can look at all the different aspects in your life and think, all right, you know, everything's balanced and doing really well. Then you're flourishing. You're doing great. But if they're something's off balance then you get to work on those particular things, and that's still flourishing, but you're you're flourishing in the matter of the fact that you're working on bettering those different components. So that's kind of what flourishing means to me. | Still flourishing even if working on bettering different areas of flourishing | Feelings of flourishing | Whole person health |
| 3 | Flourishing | For a personal definition, it when I think about if I feel like I'm flourishing a couple things are, I don't find that A lot of my like mind space is taken up on worrying about things as much. | Feeling mentally spacious, not worrying | Feelings of flourishing | Whole person health |
| 3 | Flourishing | And then also I feel like I can bounce back from things quicker | Feelings of overcoming difficulties in life | Feelings of flourishing | Whole person health |
| 3 | Flourishing | . . . and then also I notice that if I'm more focused on art, creating something as you usually when I feel like I'm flourishing either because it makes me feel like I'm flourishing or I am flourishing and thus I do more art not sure which one is which but those are kind of my personal like definitions I guess I would say for myself. | Feeling creative | Feelings of flourishing | Whole person health |
| 3 | Flourishing | Yeah, and to me it's like a step above that state of like everything being okay when we talk about domains of well-being or dimensions of wellness. . . So the word flourishing seems to be like a step above just everything's okay. It's like you're growing. | Feelings of thriving | Feelings of flourishing | Whole person health |
| 3 | Flourishing | okay when we talk about domains of well-being or dimensions of wellness. But like the idea that. You can really drive toward what brings you meaning and purpose. . . Doing the things you were meant to do. | Meaning and purpose | Key domain of flourishing | Whole person health |
| 3 | Flourishing | Yeah, I would agree with that when I think of flourishing, I think of like you're shining, you're glowing like things are really going well in areas that are important to you. | Feeling of exceeding one's potential as a continuum | Feelings of flourishing | Whole person health |
| 3 | Flourishing | For me, I think, the, component of (inaudible), and that you're really. It's similar to what (participant name) said, like. You're not just okay. You're kind of exceeding your expectation of okay. | Feelings of thriving | Feelings of flourishing | Whole person health |
| 3 | Flourishing | And, even if you're working towards something, that it. You feel like you're doing that in a really good way and you're feeling like you're successful at the goals that you're setting and the progress you're making towards whatever it is that you're doing. | Feeling of exceeding one's potential as a continuum | Feelings of flourishing | Whole person health |
| 3 | Flourishing | (Participant name) made me think when you said that thriving, not surviving. [You're not just okay. You're kind of exceeding your expectation of okay.] | Feelings of thriving | Feelings of flourishing | Whole person health |
| 3 | Flourishing | Lot of us are in survival mode but thriving, being successful, not just hanging in there. So. | ? | ? | ? |
| 4 | Flourishing | I feel like if you are completely balanced within yourself, you can resonate that out to other people and make them feel the same balance that you feel within yourself. So it's kind of like reciprocal. If you are able to find it within yourself, you may pass that feeling on. | Feeling completely balanced | Feelings of flourishing | Whole person health |
| 4 | Flourishing | . . . you can resonate that out to other people and make them feel the same balance that you feel within yourself. So it's kind of like reciprocal. If you are able to find it within yourself, you may pass that feeling on. | Reciprocally radiating complete balance in to others | Feelings of flourishing | Whole person health |
| 4 | Flourishing | I feel flourishing is, when things in your life are in a balance. Where it's not stressful. I guess like and maybe not like, well I aint gonna say stressful cause you know, just living is stressful but like you know the eustress, the good stress, versus the negative stress. | Feeling completely balanced | Feelings of flourishing | Whole person health |
| 4 | Flourishing | And normally, it's like if your finances are balanced . . . | Financial and material stability | Key domain of flourishing | Whole person health |
| 4 | Flourishing | . . . if, like your relationships are healthy. | Close social relationships | Key domain of flourishing | Whole person health |
| 4 | Flourishing | You're meeting your goals. | Meaning and purpose | Key domain of flourishing | Whole person health |
| 4 | Flourishing | The, you know, the foods you're eating make you feel good so you're not in pain or any thing going on. | Feeling well from nourishing foods | Feelings of flourishing | Whole person health |
| 4 | Flourishing | I missed that, sorry. Flourishing is having positive internet connections haha. No, having positive connections in your life, you know. | Close social relationships | Key domain of flourishing | Whole person health |
| 4 | Flourishing | Forward progress in you know, both your physical well-being, your emotional well-being, your career, your relationships. You're flourishing, you're growing and you're moving through things and not having stunted growth, you know, that you're able to get through things and move on to the next thing. And to the next step in your in your life and your career and your relationships. | Feelings of thriving | Feelings of flourishing | Whole person health |
| 4 | Flourishing | And just overall having the energy to commit to the things that you are important in your own. | Feeling energy / ease in daily life | Feelings of flourishing | Whole person health |
| 4 | Flourishing | You know, I may not have the same expectation of all of those domains as someone else, but in my own personal viewpoint of the world, I have enough to put towards each of those places for myself or I'm not coming from a place of. Oh, here's one more thing I have to do and being resentful. Instead, it's coming from a, you know, this is an opportunity that I get to be able to do these things. | Feelings of thriving | Feelings of flourishing | Whole person health |
| 4 | Flourishing | I think of thriving. And following up on what other people said it's kinda like working from their overflow. Yeah. I think (participant name) said that, yeah. | Feelings of thriving | Feelings of flourishing | Whole person health |
| 1 | Newsletters | I like the layout . . . | Likes layout | Layout is good | Newsletter layout |
| 1 | Newsletters | . . . however I feel like it's too many words. | Too many words | Amount of information | Newsletter information |
| 1 | Newsletters | And I was gonna, I was gonna say I like if it's a newsletter. You kind of know that it is gonna have a good bit of words, but I like that it is blocked in each section. So. Even though it's a lot of words for one, you know, for a lot of, for one page. | Appropriate amount of words | Amount of information | Newsletter information |
| 1 | Newsletters | At least it is broken up into different sections and it doesn't take long to read each little section. | Content is broken up into sections | Layout is good | Newsletter layout |
| 1 | Newsletters | And I still, I still like the warm colors, the calming colors. I still like that. | Likes warm, calming colors | Colors are desirable | Newsletter colors |
| 1 | Newsletters | I feel like it's a great balance of blocks and circles. I don't know the circles really are eye catching. I, well, I think it's well done. | Balanced shapes and pictures in layout | Layout is good | Newsletter layout |
| 1 | Newsletters | I actually had the opposite reaction of the words. I was like, thank God it's not as much words as we normally have to read. I think just because. I don't know about anybody else, but I read a lot of research, so. I thought that it was kind of balanced that it's only a paragraph. Or just a couple of sentences under each section. And I do like that there. | Appropriate amount of words | Amount of information | Newsletter information |
| 1 | Newsletters | Multiple pictures, like (other participant) says it seems balanced. | Balanced shapes and pictures in layout | Layout is good | Newsletter layout |
| 1 | Newsletters | I have a question. Is it purposely, purposely, like, running back, six things. Is it's supposed to be something that relates to. Each of those 6 domains (of flourishing) you talked about every week. | ? | ? | ? |
| 1 | Newsletters | I've been going back and forth. So the sources box. I don't know why I don't necessarily love the color choice (brown). I don't know. I almost think it needs to maybe stick with that blue if that's. Or I don't. I'm just torn. I don't know. I don't know if it needs to be highlighted like that. That's what I'm saying is like there's these beautiful colors of blue and I just feel like it kind of sticks out and I don't know if we're wanting it to stick out like it is. Or if it's just there. We just kind of want it to blend in. I don't know. I thought I would share it. | Compatible colors | Color suggestions | Newsletter colors |
| 1 | Newsletters | I like ease of use. I, you said it complements the online sessions or Zoom sessions. So it's not necessarily another job or another something else for us to do. If we don't read it, I like that is it provides you with the summaries of. They said in health settings or we learn it and health settings. Having a resource or a takeaway, I always compliments the lesson. And so I like that part about it. I don't feel. Oh, really stress that this is something else that I have to do like another component of participating. I feel like it complements the thing that we're already doing. | Likes newsletters as a takeaway to complement weekly sessions (not an addition) | Desirable resources | Newsletter information |
| 1 | Newsletters | I would personally do this. I think what's gonna hold any back any other agents back is just the timeframe and you know they're gonna have to hold themselves accountable or logging in and and doing the work. As with anything, it's just the accountability of it. | Resistant views to pausing / taking a break for self-care | Individual barriers to participating in yoga practices | Considerations for implementing wellbeing program |
| 1 | Newsletters | Maybe if it was say a hybrid, the lessons are sent to an early along with the newsletter and then because this is what I have to do with my Walk With classes. I have to give the lessons. Before we get out there. So that when they get out there they're ready to we're ready to start playing and working and being active. It's just something that I have to do because I know if I come out there. With less than and hang in. They're already over it. They've been at work all day or they're ready to get to work or they they're just they just wanna be active. They don't wanna hear necessarily. Take the time to be in that lesson beforehand. I just want to have that activity. So I send them the material. Prior and they watch it or in and do them. Quiz or whatnot before they even go out there to the act of activity. | Hybrid asynchronous lectures with synchronous activity | Suggestions for FLEX program | Wellbeing program perceptions |
| 2 | Newsletters | I like how the information is broken up into readable and visual chunks. I like that. | Content is broken up into sections | Layout is good | Newsletter layout |
| 2 | Newsletters | I might change the dark purple to be more in line with, that slate blue. I just feel like it's competing too much with the color . . . | Compatible colors | Color suggestions | Newsletter colors |
| 2 | Newsletters | I really like I really like, again, the plain font, the simplicity of the font . . . | Likes accessible font / font hierarchy | Font | Accessibility of newsletters |
| 2 | Newsletters | (I really like I really like) the, text hierarchy where you have the titles larger. | Likes accessible font / font hierarchy | Font | Accessibility of newsletters |
| 2 | Newsletters | I like the coloring. For the most part. We can talk about that later. | Likes colors | Colors are desirable | Newsletter colors |
| 2 | Newsletters | I agree with (participant). [I like how the information is broken up into readable and visual chunks. I like that.] | Content is broken up into sections | Layout is good | Newsletter layout |
| 2 | Newsletters | I agree with (participant). [I might change the dark purple to be more in line with, that slate blue. I just feel like it's competing too much with the color . . .] | Compatible colors | Color suggestions | Newsletter colors |
| 2 | Newsletters | I agree with (participant). [I really like I really like, again, the plain font, the simplicity of the font, the, text hierarchy where you have the titles larger.] | Likes accessible font / font hierarchy | Font | Accessibility of newsletters |
| 2 | Newsletters | I agree with (participant). [I like the coloring. For the most part. We can talk about that later.] | Likes warm, calming colors | Colors are desirable | Newsletter colors |
| 2 | Newsletters | I really like your logo up here too. I like how it says "FLEX" on the circle . . . | "FLEX" on logo more clear | Logo more clear | FLEX logo perceptions |
| 2 | Newsletters | . . . and it helps me understand now why on the poster (recruitment flyer), the globe, the icon for holistic health. I think that's what that is. I understand now why that is not so prominent in your logo because it's easier to see it in this logo. | More clear that globe on logo represents holistic health | Logo more clear | FLEX logo perceptions |
| 2 | Newsletters | I noticed how the Flourishing and the Extension you do have those in bold on the newsletter. So that's what we were talking to. They're talking about that (is a good thing). I do like that. I like it better than the underline. Yeah. | Bold instead of underline FL EX in Flourishing in Extension | Font | Accessibility of newsletters |
| 2 | Newsletters | I agree with that. [bold better than underline for F L E and X in Flourishing in Extension) | Bold instead of underline FL EX in Flourishing in Extension | Font | Accessibility of newsletters |
| 2 | Newsletters | Can I ask a question about the flyers (newsletters)? Okay, the circular images that you have. Are you going to run out of circles? Like are you going to run out of things to showcase? Like I'm looking at the one next to "Creating Creativity" and I'm like what is that? Is that a sunburst? Is that like a close-up of an eye? I don't know. I took my kids to the eye doctor. So that's something that's what was on my brain. . . and maybe it's because it's creativity, you're going with abstract and that's fine too. . . And maybe it's just that one image that I should not worry about it. | Abstract images not desirable | Undesirable imagery | Newsletter imagery |
| 2 | Newsletters | I like the one above it. Of the people who are doing the stretches. I think that correlates really well . . . | People doing yoga | Desirable imagery | Newsletter imagery |
| 2 | Newsletters | Mimic you have that repetition going on throughout the newsletter with the logo and the the images that you're using. I do like that. | Balanced shapes and pictures in layout | Layout is good | Newsletter layout |
| 2 | Newsletters | I understand what (participant name) was saying about the circle and the "FLEX" (the geometric shape in FLEX logo). But still I don't know how many people know that is about. What you mentioned, (participant name). The circle was. . . About All the things. I don't know what is the meaning of the circle. I mean, where it says "FLEX". The little over the book (logo). | Not clear that globe on logo represents holistic health | Logo unclear | FLEX logo perceptions |
| 2 | Newsletters | I think, I think. Balancing. I think in "Sources", maybe that could be a background , or a little darker so the white can pop up or I don't know if it's white or page, but if it's, I would do a white. The thing is, so it comes up, you know, it pops up more. | Blues instead of brown | Color contrast | Accessibility of newsletters |
| 2 | Newsletters | Mostly just make sure it's accessible. Per the rules by 2026. |  | Accessibility important | Accessibility of newsletters |
| 2 | Newsletters | I agree with having the sources that, flesh colored box. There's not a lot of contrast between that color and the white. I would almost change it to the same color of blue that the QR code is in. | Blues instead of brown | Color contrast | Accessibility of newsletters |
| 2 | Newsletters | The bright indigo is kind of competing with the muted slate blue. If you wanted to incorporate. More of like the flesh color that you have your sources in. Yeah, never mind. I'm gonna backtrack on that. But I still stand by the color of the font and the titles, the subtitles. They're too intense to go with the muted tones of your heading of your header. In my opinion. . . I'm talking about where it says the "Subtle Anatomy" and then the paragraph under it and my screen it's showing up is like a dark indigo blue which is competing with a more muted tone of the header. It's they're just 2 different colors of blue. And so I, unless it's a, I don't know, I feel like it just doesn't go with your branding in your logo to have that bright indigo. I would stick with the more the darker color. So if you can get your color dropper and pick the color of the the foreground in your header and get that darkest color and maybe go with that. Does that make sense? Then it just wouldn't be so. Extreme I feel like. | Compatible colors | Color suggestions | Newsletter colors |
| 2 | Newsletters | I understand what you say (about colors). I wanted sometimes what I do I use a website called color hexa. And then I pick the hex number of my main color. And then that way I can see shades that are kind of compatible. And that helps a lot. | Compatible colors | Color suggestions | Newsletter colors |
| 2 | Newsletters | Are you hoping that your color scheme will coordinate with Virginia Extension your logo with the burgundy? | Coordinating with Virginia Extension logo? | Color intention? | Newsletter colors |
| 2 | Newsletters | So are you wanting this? Are you wanting to stick with the more violet, blue color, are you wanting it to be more subdued? | Compatible colors | Color suggestions | Newsletter colors |
| 2 | Newsletters | So when we get this newsletter. Would it be it would be a 1 page newsletter? Okay, cause I'm seeing like front to back newsletter or 2 pages. Now that I'm looking at them side by side, I'm like, oh, that's a lot of circles. That's a lot of movement on the page. That's a lot of repetition. There's a lot going on, but I think it's I think it's probably okay if you just are getting one page. | One page is good | Layout is good | Newsletter layout |
| 2 | Newsletters | I understand what you say, (other participant name). Sometimes It's good to have a different. Different composition on the other page So then it also helps people to find information. Is not like paragraph, circle, paragraph circle, everything and then where is where I saw the blah blah blah blah blah. So, So that I think that will help. But I don't know if that will change. | One page is good | Layout is good | Newsletter layout |
| 2 | Newsletters | Will this be like maybe in the email or as a an attachment or a combination of those? |  |  |  |
| 2 | Newsletters | I would even suggest, I don't know how your team feels about this, but I don't think both pictures are necessary. If you wanted to remove one of them, I think it would still look appropriate. Like I would still get a feel for, you know, I'm thinking about happiness habits and that includes exercise and. So I don't know that you necessarily need both. . . And the other thing is that maybe if. Let's say for me, I will eliminate the yellow circle (image). And move that paragraph to the left and then I can create a bigger image (of people doing yoga). | Fewer images as an option | Undesirable imagery | Newsletter imagery |
| 2 | Newsletters | will say I appreciate that you're not using like cartoon graphics and stuff like that. | Cartoon graphics not desirable | Undesirable imagery | Newsletter imagery |
| 2 | Newsletters | I like that it's a more, realistic perception of what will be happening. So good job. | People doing yoga | Desirable imagery | Newsletter imagery |
| 2 | Newsletters | So it's not just one person that you see is other people. So they're different kind of people, they're in that image. And I said that because everything (each image) is single, right? One footprint. One guy, one pair of hands, so. They just gives kind of. A different style. | Preference for imagery of people in groups | Desirable imagery | Newsletter imagery |
| 2 | Newsletters | Well, I was thinking also if the more images you have, I think sometimes the larger is the PDF. So. Most of it, I, we don't have problems in North Carolina if we have our, you know, Extension Apple laptops, but let's say in some counties. They don't have the Apple laptop. They have the county PCs and then they may be older PCs. So. Those considerations also you may want to think about. | Too many images will make pdfs too large for some computers | Considerations for emailing newsletter | Accessibility of newsletters |
| 2 | Newsletters | I'll add, too, Frazier that our university has started blocking images and so like when I got your email, all I get is like a little icon with like a "Warning!" And I have to like go through this process and maybe your universities are the same. | Email blocking images | Considerations for emailing newsletter | Accessibility of newsletters |
| 2 | Newsletters | I wondered, another thought, is have you have you played around with . . . but have you played with the idea that it is justified to where the words aren't ending in such a random place is that does that make sense? . . . If you took out the "Creating Creativity" or yellow circle and made it justified so the words so that their space so they do end in block form and maybe that's just a personal preference. And, and maybe you've already played with that and this looks better. Just a thought. I'll stop commenting. | Font justified / formatted so words do not end in random places | Font layout | Newsletter layout |
| 2 | Newsletters | For example, and maybe this might Maybe it is not a big deal, but like the happiness habits, I would have it justified to be curved along that picture. I think visually that will look nicer. | Curving font headings around images | Font layout | Newsletter layout |
| 2 | Newsletters | -- I like that you haven't centered everything everything is left justified -- | Left justified font, not centered | Font | Accessibility of newsletters |
| 2 | Newsletters | We're giving you so many ideas, aren't we? Lots of a different ideas. And I just wanted to throw this out and, Yeah, maybe you've already mentioned this and I missed it, but the brown at the bottom, if you could incorporate that, if you wanted to leave the pictures. Maybe incorporate that color and put a border behind the pictures and that would make it stand out. And then it would kind of make that color flow up to the top and all throughout the flyer. . . but, but if you have, you could incorporate that brown up in the top somewhere maybe behind the circle that it kind of make it offload. | Compatible colors | Color suggestions | Newsletter colors |
| 2 | Newsletters | I'll throw out that, that I like the pictures personally and the, starburst (image) or whatever that is. I think I might choose a different picture other than that one because it's not very eye catching. | Abstract images not desirable | Undesirable imagery | Newsletter imagery |
| 2 | Newsletters | love the man (image of person doing yoga). I love that one. | People doing yoga | Desirable imagery | Newsletter imagery |
| 2 | Newsletters | . . . and then that other flyer - The the foot (image of footprint) I think I wasn't super crazy about that one . . . | Imagery of feet not desirable | Undesirable imagery | Newsletter imagery |
| 2 | Newsletters | And, but it, but like I say, if you, you know, and I love the hands | Hands with sun is desirable | Desirable imagery | Newsletter imagery |
| 2 | Newsletters | I would say yes (interested in FLEX from newsletter). One thing that, I don't know if you would have space for that, maybe at the, at the bottom (of the newsletter). Yeah, a reminder, "Don't forget, log in la de da de da next week" or you know, a reminder of that kind. | Reminder about weekly session | Content suggestions | Newsletter information |
| 2 | Newsletters | I like that it gives a preview for that week so I can be contemplating or thinking about on my drive home. Okay, we're talking about rooting, not ruminating. What does that mean? How can I apply that? Not only at work, but in at home. So I have this work life balance. I like the little snippets. I think the overall idea of the newsletter adds to the programming because it's also something I can refer back to if I don't wanna like rewatch the entire Zoom session again. It's something I could take a screenshot of if I like that thought or quote and tape it up on my pinboard and say, okay, this is something I'm going to focus on and improve on. I like that. | Likes newsletters as a takeaway to complement weekly sessions (not an addition) | Desirable resources | Newsletter information |
| 3 | Newsletters | I like the "Rooting, Not Ruminating" title . . . And I think some ag agents may also like that ruminating language and understand that and it brings that image up of kind of chewing on your thoughts, which I think about a lot when we're like over and over. It's like. | "Rooting, not ruminating" | Message is desirable | Newsletter information |
| 3 | Newsletters | . . . then the "How: is like just that idea of taking a short break and walking outside. I think that simple and accessible and taking a breath. | Information on simple, accessible practices is desirable | Desirable resources | Newsletter information |
| 3 | Newsletters | I like that the logo at the top has FLEX over the circle. I think that looks nice. | "FLEX" on logo more clear | Logo more clear | FLEX logo perceptions |
| 3 | Newsletters | And yeah, overall, I like (the practice in the newsletter). I think it it seems easy. | Information on simple, accessible practices is desirable | Desirable resources | Newsletter information |
| 3 | Newsletters | And the colors are even calming. The tan, the blues, or greys, whatever. | Likes warm, calming colors | Colors are desirable | Newsletter colors |
| 3 | Newsletters | Yeah, I think it's really balanced. It looks really good. With white space, the pictures, the the way that things are highlighted. | Balanced shapes and pictures in layout | Layout is good | Newsletter layout |
| 3 | Newsletters | Frazier is that that (non discrimination) statement just got updated. So. That's a, a, a, an old (non-discrimination) statement. But that, just, so you know, we do have a new one. | Non-discrimination statement | Cooperative Extension content | Newsletter information |
| 3 | Newsletters | I'm wondering about. So I noticed that you bolded, "subtle anatomy" (in-text). And so then I was thinking, okay. Rooting and ruminated and stability (in-text) would be bolded as well. But they're not so that I was just wondering what made you choose that. | Consistency with bolding key words in-text | Font layout | Newsletter layout |
| 3 | Newsletters | And then I like that you include the sources here because they're always helpful. I was just wondering like, they (citations in-text) just feel a little big. And I didn't know if you could do like a footnote and so like, you know, like the little number up top | Superscript instead of parentheses | In-text citations | Newsletter citations |
| 3 | Newsletters | I agree with the, numbers (citations in-text). Right, smaller subscripts instead. | Superscript instead of parentheses | In-text citations | Newsletter citations |
| 3 | Newsletters | The, I like how the FL and the EX in "Flourishing Extension" is bolded instead of underlined in this one. | Bold instead of underline FL EX in Flourishing in Extension | Font | Accessibility of newsletters |
| 3 | Newsletters | And looking at the images, the only image that I think certain program areas like in particular might not be as attracted to is the first image with the hands and sun and everything. . . As opposed to images that do tend to be. Some people may see them more as like spiritual than nature. And they might take issue with it I guess. I mean, that's not all Extension agents, but do you think some of them . . . | Hands with sun not desirable | Undesirable imagery | Newsletter imagery |
| 3 | Newsletters | The more that we can kind of like stick to more natural pictures where it's just like a flower or something like that. | Nature imagery | Desirable imagery | Newsletter imagery |
| 3 | Newsletters | Like, if you're wanting to invite them to participate, it might be. Similarly to the other comment, with the (recruitment) poster. Having a link that could be in text. Okay. Would be helpful. | Add in-text link in addition to QR code | Considerations for emailing newsletter | Accessibility of newsletters |
| 3 | Newsletters | And I think it's a good amount of words. I don't think it should be more (words) than this. But if you can lessen it even more and maybe just have links to articles that could be helpful too. | Appropriate amount of words | Amount of information | Newsletter information |
| 3 | Newsletters | I'll add that I do like the fact that you, are going to have a QR code or a link to videos. It just kind of makes this more than just a written document, which is nice. | Likes link / QR code to more resources / practices | Desirable resources | Newsletter information |
| 3 | Newsletters | I'm just gonna say like the. The "Subtle Anatomy" is, could be a little bit off-putting to people who don't have that as a personal kind of understanding and belief that and you know just kind of looking at chakras can be a little too woo-woo when we're talking about like really practical ways to support well-being. So just that being like. Right at the top there week two it. I feel like the subtle anatomy is a pretty subtle thing to understand. | Remove chakras | Content suggestions | Newsletter information |
| 3 | Newsletters | Yeah, I like that last paragraph. And now that I see it, like to understand like how you're making it flow. I do like that's consistent like that middle part of the "what" and "how". I know, I know you're asking like, what do you like and what do you don't like? Sorry, I'm just kind of mixing them together. | Content is broken up into sections | Layout is good | Newsletter layout |
| 3 | Newsletters | And then I'm wondering, just based on what was said about, you know, starting with subtle anatomy. I mean, I don't, I don't know if you have a, like I'm imagine. Hmm. I'm imagining you have a specific way of, you know, organizing this. But you know, maybe like since it's a 9 week program like earlier in the weeks. You start with something that's a bit more softer and easier to understand and then get to something like subtle anatomy. Cause I do think like "happiness habits" or something that would be just like, "oh yeah, like, you know, I care about happiness" and so that would be really easy for someone to like get into and grow from there. | Remove chakras | Content suggestions | Newsletter information |
| 3 | Newsletters | Do the sources have to be on this? Or could it be a link where the source that you just have a reference point to all the sources for the entire time. And if somebody wants to see the specific sources, they can click that link and then the sources come up that would save you a lot of space on the flyer and kind of help space it out even more. I like I just think of when I receive things it's not that I'm not interested in it, and if I'm even if I'm choosing to participate in some stuff like there's so much information coming at us constantly. That in some ways it's a teaser that link to something else can be helpful, but I do, it is still very brief. | Link to sources instead of listing on one-page newsletter | Suggestions for sources section | Newsletter citations |
| 3 | Newsletters | We here, I'm at Penn State, and I've had to do a poster presentation and the sources took up so much of the poster, we actually put a QR code on the poster presentation. So, you know, that you get the credit and the references and the resources or whatever, but it doesn't. And again, I think that's a, if you're allowed to do that, I know (participant name), we probably would not be allowed to do that in a publication we do here at Extension, but I've seen it done on like professional posters for conferences and stuff just to save space, but. If you're looking to get rid of that section to make room for the other stuff. But. | Link to sources instead of listing on one-page newsletter | Suggestions for sources section | Newsletter citations |
| 3 | Newsletters | Or could it be on the back, right? Even though the back would be a page too, if it's a digital thing, like could there be a page two to kind of with the you know, with (non-discrimination, education only) statements and the source and. I don't know. | List extra information on second page / back page | Layout suggestion | Newsletter layout |
| 3 | Newsletters | I know the (non-discrimination) statements a lot of times have to be on the front. They're special rules and there might be special rules for the references. Again, I don't know them, but. | Non-discrimination statement | Cooperative Extension content | Newsletter information |
| 3 | Newsletters | But again, if this audience is Extension professionals. We all know that stuff has to be on there. So I don't think it would bother us as Extension as much as it would the general public. Like I think the general public does not want to see all that crap, but Extension people, we know that's it's scholarship, it's scholarly, it's research-based. So | Scholarly, research-based information okay for agents as audience | Cooperative Extension as audience | Newsletter citations |
| 3 | Newsletters | So what if like if this is only gonna be digital and not printed couldn't it just be longer? The page be longer to move that. The stuff down a little? Give a little more space, I don't know. | Increase length of document to make more spacious | Layout suggestion | Newsletter layout |
| 3 | Newsletters | [Size of newsletter] Instead of like an 8 and a half by 11 like longer like 17. | Increase length of document to make more spacious | Layout suggestion | Newsletter layout |
| 3 | Newsletters | [Size of newsletter] Likes 11 by 17 or yeah. | Increase length of document to make more spacious | Layout suggestion | Newsletter layout |
| 3 | Newsletters | (Longer file size) So it's spread out and then the stuff's on the line. | Increase length of document to make more spacious | Layout suggestion | Newsletter layout |
| 3 | Newsletters | I mean, personally, I think if you are demonstrating throughout like you already are that there are sources connected with it and that it is coming from sources. And you like make it clear that there is a way to get to this or somebody wants to dive deeper and to me as a Extension professional I'm seeing it's research based and I know how to get to see them, but most of us aren't gonna take the time to look at the sources unless we really wanna dive deeper. So. I mean. To me it accomplishes it. Even if you just have some sort of link. And it cleans up the poster a little bit because you can't not have this number at the very bottom. | Link to sources instead of listing on one-page newsletter | Suggestions for sources section | Newsletter citations |
| 3 | Newsletters | Lot of for our webinars a lot of times we put the references at the back and now for a while we have them on the PowerPoint which I personally thought was awful for the public because it was all cluttered and now and (participant name) I don't know if you know if we're going back I don't I don't keep up with that but I think The clutterness, like you have to have them, I totally agree. Just a matter of, you know, putting them on there, but. | Link to sources instead of listing on one-page newsletter | Suggestions for sources section | Newsletter citations |
| 3 | Newsletters | I personally like the idea of the QR code because if I did want to go check out the resources, you could just, or even, If that QR, if some link was clickable that took you to a Google document or Outlook or whatever Microsoft document that had all the resources listed on there for you to check out if you wanted to delve in more. But outside of that, yeah, I don't know that. Because we do our Buzz Body invites newsletter. And we have a resource section. But it's only like three links, if anything, that we're referring to during that newsletter. So. | Link to sources instead of listing on one-page newsletter | Suggestions for sources section | Newsletter citations |
| 3 | Newsletters | And I understand this is a research project and there's a lot of stuff, but like I said, that's only time I've ever really seen it done was on a professional poster at a conference and my coworker and I were trying to we couldn't fit everything on the poster and so we got you know and it worked I mean the people who wanted it they're like because again it was at a conference. They just put their phone up to get the references for the poster but again, some institutions have rules about that. Like I said, we were at a conference. I don't know if here at Penn State we would get away with that in a webinar or something like that or handout, but it was a presentation. So. | Link to sources instead of listing on one-page newsletter | Suggestions for sources section | Newsletter citations |
| 3 | Newsletters | And another thought that I have is if you can link to the sources, then you could just have one page or document that has all the sources for all the newsletters. And then if you're reusing them, you just use the same number all the time and then you don't have to like format it for everything on the newsletter. But yeah, I don't know. That all comes down to whether or not you can. Virginia Tech is going let you do it that way. I guess. | Sources listed all in one place for all newsletters for linking each week | Suggestions for sources section | Newsletter citations |
| 4 | Newsletters | I like the categorization. Like the "happiness" and "creativity" and the "long lasting habits". | Content is broken up into sections | Layout is good | Newsletter layout |
| 4 | Newsletters | I like the positioning of the pictures and that there is a staggered. Yeah, to start. | Balanced shapes and pictures in layout | Layout is good | Newsletter layout |
| 4 | Newsletters | I really like the second one that you have a "how", which is a call to action. I could see doing that for each section if they're separate. | Information on simple, accessible practices is desirable | Desirable resources | Newsletter information |
| 4 | Newsletters | I like the way you balance the circles, the top, the right, the bottom. | Balanced shapes and pictures in layout | Layout is good | Newsletter layout |
| 4 | Newsletters | I really don't like the yellow (image). I don't know why, but I don't like it. I wish I had a concrete reason to give you other than I keep. it's definitely drawing my eye to that and not the other than I keep it's definitely drawing my eye to that and not the other things. It's definitely drawing my eye to that and not the other things. | Abstract images not desirable | Undesirable imagery | Newsletter imagery |
| 4 | Newsletters | I'm not sure why the reference numbers are bolded. | Do not bold in-text citations | In-text citations | Newsletter citations |
| 4 | Newsletters | I wouldn't put any more text on white like that. I think that's the maximum amount of text that should go in there. | Appropriate amount of words | Amount of information | Newsletter information |
| 4 | Newsletters | So that's okay. You do have kind of the logos and the disclaimer statements there I think that's important. | Logos important | Cooperative Extension content | Newsletter information |
| 4 | Newsletters | So that's okay. You do have kind of the logos and the disclaimer statements there I think that's important. | Non-discrimination statement | Cooperative Extension content | Newsletter information |
| 4 | Newsletters | And I do like that the the images are brighter . . . But I do like that you've got some brighter images . . . I know we have to put all the disclaimers and stuff but I agree with that but I do like the, more colorful images, more light. | Desires brighter colors | Color suggestions | Newsletter colors |
| 4 | Newsletters | . . . but I agree with you that one big yellow. It's a little distracting. . . But that one is a bit bright. Abstract supposed to be clouds or sun or creative? | Abstract images not desirable | Undesirable imagery | Newsletter imagery |
| 4 | Newsletters | You got a little more inclusive images. | Inclusive imagery | Desirable imagery | Newsletter imagery |
| 4 | Newsletters | I like the topics, the concepts . . . | Flourishing themes and yoga concepts | Message is desirable | Newsletter information |
| 4 | Newsletters | . . . but I do believe it's a lot of words. I know we have to put all the disclaimers and stuff | Too many words | Amount of information | Newsletter information |
| 4 | Newsletters | And (the yellow image is) so much larger that it than the other 2 circles. It doesn't feel balanced. So maybe that's why. | Make circular images the same size | Layout suggestion | Newsletter layout |
| 4 | Newsletters | We're all staring right there thinking. It's it has a lot of real estate. [about yellow image] | Make circular images the same size | Layout suggestion | Newsletter layout |
| 4 | Newsletters | It's illuminating. It's a "creativity". Haha. [about yellow image] | Abstract images not desirable | Undesirable imagery | Newsletter imagery |
| 4 | Newsletters | So maybe that's why. I guess the, and I was going to say something about the colors. The, on the top, the blue green. I know it's mountains, I still see that the backdrop is in the mountains, but the I don't know it's a little dark for me. It's a little dark. And I, and I only notice mountains because I went up a little closer, but the initial. Well, it was just kind of, yeah, look stormy. Yeah, I like that description. It looks stormy. | Compatible colors | Color suggestions | Newsletter colors |
| 4 | Newsletters | And I think the sources have a place. I guess I don't know. It's kinda like. I don't know if they go somewhere else. Cause I know, you know, we want to have access to where the sources from, but I don't know if. Like it it can go out in the email with the newsletter and not necessarily on the newsletter maybe. Cause I would like more room for the meat of what the program . . . | Link to sources instead of listing on one-page newsletter | Suggestions for sources section | Newsletter citations |
| 4 | Newsletters | [And I think the sources have a place. I guess I don't know. It's kinda like. I don't know if they go somewhere else.] Right, maybe make it 2 pages. I think that's a good idea too. | List extra information on second page / back page | Layout suggestion | Newsletter layout |
| 4 | Newsletters | You know, for VCE, we have to have that nondiscrimination statement. So, if you made it 2 pages, you could put those things on the back. | List extra information on second page / back page | Layout suggestion | Newsletter layout |
| 4 | Newsletters | I do like it looks like you're gonna put in a link for videos. I always like that. | Likes link / QR code to more resources / practices | Desirable resources | Newsletter information |
| 4 | Newsletters | So I like the FLEX logo here. Better than the one on the first page because it's says "FLEX" like that to me makes more sense that the logo for the program. | "FLEX" on logo more clear | Logo more clear | FLEX logo perceptions |
| 4 | Newsletters | But I keep trying to understand the relationship between the globe and the book and health and happiness. And it's a nice logo like I like it and if it was for a library I'd be like oh that makes perfect sense. But I don't know that the logo makes sense to me. So I feel like we're kind of nit picking and going through all sorts of little things, but I just. I don't know, I keep staring at the logo from the first one. To this one and the other one before this because I was late but Yeah. | Not clear that globe on logo represents holistic health | Logo unclear | FLEX logo perceptions |
| 4 | Newsletters | I think one of the aspects of the FLEX is journaling so that may be where the book (in the logo) comes into play. | Not clear that book on logo represents journaling / self-study | Logo unclear | FLEX logo perceptions |
| 4 | Newsletters | I think one of the aspects of the FLEX is journaling so that may be where the book (in the logo) comes into play. | Not clear that book on logo represents journaling / self-study | Logo unclear | FLEX logo perceptions |
| 4 | Newsletters | Yeah, when I first saw (the FLEX logo), I would almost, I think I've seen like a global studies logo that looks a bit like that. So it's like the book means study . . . | Not clear that book on logo represents journaling / self-study | Logo unclear | FLEX logo perceptions |
| 4 | Newsletters | [Yeah, when I first saw (the FLEX logo), I would almost, I think I've seen like a global studies logo that looks a bit like that. So it's like the book means study] and the globe means, um, I don't know. I could see. Like a person with the FLEX globe on their hand or something. I don't know. | Not clear that globe on logo represents holistic health | Logo unclear | FLEX logo perceptions |
| 4 | Newsletters | Yeah, anyhow, I do like the FLEX concept and idea. | Flourishing themes and yoga concepts | Message is desirable | Newsletter information |
| 4 | Newsletters | I feel like the globe (geometric shape in logo) gets lost here a bit | Globe on logo gets lost behind "FLEX" | Logo unclear | FLEX logo perceptions |
| 4 | Newsletters |  |  |  |  |
| 4 | Newsletters | I find it hard to know what are. What do you want the people to do with the newsletter? Is there like in this week focus on these actions or, you know, is there a space for journaling or tracking or is this just a general information that "Oh, that's good to know." You know what I mean? | Clarity that newsletter is an educational resource | Content suggestions | Newsletter information |
| 4 | Newsletters | I guess I would also say going back to the first page, be careful if you have images where the man's arm is blocking the face of the woman. Where I would be kind of, you know, cognizant of things where it looks like somebody is hogging the space of the picture, you know, what that could convey by accident. | Inclusive imagery | Desirable imagery | Newsletter imagery |
| 4 | Newsletters | I'm not caring too much for the rooting photo with the clay and the foot. | Imagery of feet not desirable | Undesirable imagery | Newsletter imagery |
| 4 | Newsletters | When I think of like rooting myself in the ground, I think of. Grass. I kind of think of a more uplifting picture, maybe. | Nature imagery | Desirable imagery | Newsletter imagery |
| 4 | Newsletters | So I think in looking at both of them, I really do feel that 2 pages would be better to have it spread out a little bit more. | Space for information to be spread out | Layout suggestion | Newsletter layout |
| 4 | Newsletters | And put the sources maybe on the back of the second page like you know and if there a specific reason that it's a 1 sided piece paper? Okay, so yeah, I would say do 2 sided because I think you could spread it out because it does It does feel cramped. I think there would be some I agree like. Absolutely with the like reinforcing some of the habits and some of the practices that you're going to be doing throughout the sessions in the newsletter so that would leave some space for that. | List extra information on second page / back page | Layout suggestion | Newsletter layout |
| 4 | Newsletters | I agree like. Absolutely with the like reinforcing some of the habits and some of the practices that you're going to be doing throughout the sessions in the newsletter so that would leave some space for that. | Information on simple, accessible practices is desirable | Desirable resources | Newsletter information |
| 4 | Newsletters | And then just the browns and the grays, like they're just not happy. So I think if you could add some more brightness to it. And spread it out. I think it would be. You know, I think you wanna have this newsletter be something that people. See and go, "oh, the newsletters here!" You know, "I'm gonna look at that and read it and reinforce the things from the class" would be my guess. So. Yeah, you wanna kinda have it. It is kind of depressing. I'm sorry. | Desires brighter colors | Color suggestions | Newsletter colors |
| 4 | Newsletters | I get subdued. I feel subdued with these colors. I think of. In marketing they have the push and the pull, right? You know, something that pushes you like motivate to do something or something that pulls at you. I don't know if these images do any of that for me. | Desires brighter colors | Color suggestions | Newsletter colors |
| 4 | Newsletters | I mean, the one with the hands and the sun is kind of inspirational, but it is it's very subdued. | Hands with sun not desirable | Undesirable imagery | Newsletter imagery |
| 4 | Newsletters | And I almost, you know, I'm on my phone, but even looking at it, it's kind of. This sounds very native. It's kind of tiring. The colors are kind of tiring for my eyes. Maybe because I'm getting older, I start to notice that. But yeah, definitely brighter. | Desires brighter colors | Color suggestions | Newsletter colors |
| 4 | Newsletters | So. I think having a rooted, you know, maybe having an image of a person in mountain pose or in a tabletop, you know. | People doing yoga | Desirable imagery | Newsletter imagery |
| 4 | Newsletters | Even if it's just somebody out standing in their grass like I can't remember who said that I agree you know when I think about walking outside showing them taking off their shoes and stepping in the grass something like that, you know. | Nature imagery | Desirable imagery | Newsletter imagery |
| 4 | Newsletters | I do like the information though talking about the subtle anatomy . . . | "Subtle Anatomy" | Message is desirable | Newsletter information |
| 4 | Newsletters | (I do like the information though talking about) rooting, not ruminating. | "Rooting, not ruminating" | Message is desirable | Newsletter information |
| 4 | Newsletters | I wish there was a way to do a simpler citation and then the same way you have a QR code . . . | Link to sources instead of listing on one-page newsletter | Suggestions for sources section | Newsletter citations |
| 4 | Newsletters | or maybe just like a little message saying for a further resources, you know, check out the bibliography here so that the people who want to can, but you're still citing effectively. | Sources listed all in one place for all newsletters for linking each week | Suggestions for sources section | Newsletter citations |
| 4 | Newsletters | I like the content. I like the theme of it. | Flourishing themes and yoga concepts | Message is desirable | Newsletter information |
| 4 | Newsletters | Yes, you know when I think about brightness and calm I think about. Sun salutations, you know, when you think about those bright earth tones. I mean, I think you went through the earth's tones here, which I like. It's just there's a little more of the the blue the brown is more of a subdued earth tone, where the reds, the oranges, the even the whites are a brighter earth tone. Does that make sense what I'm trying to say that? Yes, I think it could still be calm. But but brighter. So when you think of the rising sun, you know. It can be a sunrise, it can be very calm, but it's very bright. Same with the sunset. | Desires brighter colors | Color suggestions | Newsletter colors |
| 4 | Newsletters | Would a more tropical blue maybe work since blue is common? | Desires brighter colors | Color suggestions | Newsletter colors |
| 4 | Newsletters | I think what's throwing me too is kind of the overall palette is a mixture. It might be my screen, but the text almost seems when I'm looking at it kind of a purple, And the palette overall doesn't seem very cohesive. So maybe, you know, once you have your branding idea. I would maybe that's what's throwing it a little bit is just kind of having So, many things together that you don't, you don't see the tie in. | Compatible colors | Color suggestions | Newsletter colors |
| 4 | Newsletters | I like that image (shared in the chat). We had just used something similar in something we were doing so I had it (the image) handy. |  |  | Cultural artifact |
| 1 | Well-being | For me, it's like a rounded approach, like mind body. | Holistic wellbeing as a multi-dimensional system | Holistic wellbeing | Whole person health |
| 1 | Well-being | You know like the full picture not just as far as like healthy but mentally healthy as well. | Mental wellbeing | Dimensions of wellbeing | Whole person health |
| 1 | Well-being | I would agree. I would, like, includes in it, like, I, from, emotional . . . | Emotional wellbeing | Dimensions of wellbeing | Whole person health |
| 1 | Well-being | [I would agree. I would, like, includes in it, like, I, from] mental health . . . | Mental wellbeing | Dimensions of wellbeing | Whole person health |
| 1 | Well-being | And I mean, along with physical wellness for sure . . . | Physical wellbeing | Dimensions of wellbeing | Whole person health |
| 1 | Well-being | [I would agree. I would, like, includes in it, like, I, from] for spiritual wellness. And I mean, along with physical wellness for sure, I guess I'm also putting holistic in that just kind of encompassing all of that | Spiritual wellbeing | Dimensions of wellbeing | Whole person health |
| 1 | Well-being | I guess I'm also putting holistic in that just kind of encompassing all of that | Holistic wellbeing as a multi-dimensional system | Holistic wellbeing | Whole person health |
| 1 | Well-being | . . . where maybe things feel balanced. And even keeled. | Feeling balanced | Feelings of wellbeing | Whole person health |
| 1 | Well-being | I would tack on financial wellbeing as well. | Financial wellbeing | Dimensions of wellbeing | Whole person health |
| 2 | Well-being | I think well-being is a sense of of feeling comfortable with yourself. Physically and mentally. | Feeling comfortable with oneself | Feelings of wellbeing | Whole person health |
| 2 | Well-being | (Other participant name) liked what you had to say. I agree with that. I think well-being is being comfortable in our skin | Feeling comfortable with oneself | Feelings of wellbeing | Whole person health |
| 2 | Well-being | but also an effort to be healthy as a whole there was a discussion in our in our state last week about is holistic. Is it the type of medicine we're using or is it the whole person like WHOLE, and so we decided it was whole, the whole person . . . | Holistic wellbeing as a multi-dimensional system | Holistic wellbeing | Whole person health |
| 2 | Well-being | (whole person health . . . meaning) mental mental health . . . | Mental wellbeing | Dimensions of wellbeing | Whole person health |
| 2 | Well-being | (whole person health . . . meaning) nutrition . . . | Nutritional wellbeing | Dimensions of wellbeing | Whole person health |
| 2 | Well-being | (whole person health . . . meaning) physical health . . . | Physical wellbeing | Dimensions of wellbeing | Whole person health |
| 2 | Well-being | (whole person health . . . meaning) emotional health. | Emotional wellbeing | Dimensions of wellbeing | Whole person health |
| 2 | Well-being | And so just taking all of that into consideration and not, I would add to being comfortable in your own skin, but also understanding am I happy for the right reasons? Am I sad? Is this a situational sadness or is this a depression that's settling in? And addressing that appropriately, That sort of well being because you can still be conscious of your well-being even if you are not whole, if that makes sense, if you're still struggling with things, but if you're addressing it and you're conscious of it. And working towards improving your self in those ways. I feel like that qualifies as well. | Feeling of exceeding one's potential as a continuum | Feelings of flourishing | Whole person health |
| 2 | Well-being | I see that word (wellbeing) in my brain, and I feel like my whole body is engaged in it. So. All of my senses, and just that over arching, um, energy to get through the day. . . (Right, so just that. Wellbeing, I have, do I feel?) Yeah, so I just like I can sense it in myself as an overarching kind of word and phrase. | Holistic wellbeing as a multi-dimensional system | Holistic wellbeing | Whole person health |
| 2 | Well-being | Right, so just that. Wellbeing, I have, do I feel? Do I feel happy? | Feeling happy | Feelings of wellbeing | Whole person health |
| 2 | Well-being | (Right, so just that. Wellbeing, I have, do I feel?) Healthy? | Feeling healthy | Feelings of wellbeing | Whole person health |
| 2 | Well-being | (Right, so just that. Wellbeing, I have, do I feel?) Whole? Do I? | Feeling that everything is okay | Feelings of wellbeing | Whole person health |
| 3 | Well-being | I think well being means to me like taking care of yourself. | Taking care of oneself | Dimensions of wellbeing | Whole person health |
| 3 | Well-being | I'm trying to be a healthy version of yourself, both mentally . . . | Mental wellbeing | Dimensions of wellbeing | Whole person health |
| 3 | Well-being | [ I'm trying to be a healthy version of yourself, both] physically. Yeah, I'll just stop there. | Physical wellbeing | Dimensions of wellbeing | Whole person health |
| 3 | Well-being | And I just wanna agree with that [trying to be a healthy version of yourself, both mentally and physically.]. | Mental wellbeing | Dimensions of wellbeing | Whole person health |
| 3 | Well-being | And I just wanna agree with that [trying to be a healthy version of yourself, both mentally and physically.]. | Physical wellbeing | Dimensions of wellbeing | Whole person health |
| 3 | Well-being | I was gonna say mentally, physically, well being as an overall health of all different components. | Holistic wellbeing as a multi-dimensional system | Holistic wellbeing | Whole person health |
| 3 | Well-being | Yeah, definitely agree with that [wellbeing is multi-component including mental and physical]. | Holistic wellbeing as a multi-dimensional system | Holistic wellbeing | Whole person health |
| 3 | Well-being | I would say for me it feels like living intentionally sometimes like not just waking up and rushing into things and kind of doing, you know, just doing things that I have to or as they come it's for me while being a little bit more like, not necessarily all planned out, but I'm maybe a little slowed down and, doing things on purpose, that kind of a thing. | Ways of being | Holistic wellbeing | Whole person health |
| 3 | Well-being | I think for me there's the idea that it's being able to be in the moment . . . | Ways of being | Holistic wellbeing | Whole person health |
| 3 | Well-being | . . . having capacity and balance in different areas of life . . . | Feeling balanced | Feelings of wellbeing | Whole person health |
| 3 | Well-being | [. . . in addition to] mental. | Mental wellbeing | Dimensions of wellbeing | Whole person health |
| 3 | Well-being | Think of all the other dimensions of wellness. [You know, social, financial, spiritual and just. That feeling that everything is okay.] | Holistic wellbeing as a multi-dimensional system | Holistic wellbeing | Whole person health |
| 3 | Well-being | (Think of all the other dimensions of wellness. You know) social . . . | Social wellbeing | Dimensions of wellbeing | Whole person health |
| 3 | Well-being | (Think of all the other dimensions of wellness. You know) financial . . . | Financial wellbeing | Dimensions of wellbeing | Whole person health |
| 3 | Well-being | (Think of all the other dimensions of wellness. You know) spiritual . . . | Spiritual wellbeing | Dimensions of wellbeing | Whole person health |
| 3 | Well-being | (Think of all the other dimensions of wellness. You know) just, that feeling that everything is okay. | Feeling that everything is okay | Feelings of wellbeing | Whole person health |
| 3 | Well-being | And I'd agree with that where I look at the dimensions of wellness. So, you know, there's arguably 5, 8, whatever. And so trying to be as balanced as possible across those dimensions is overall wellness. | Holistic wellbeing as a multi-dimensional system | Holistic wellbeing | Whole person health |
| 3 | Well-being | . . . in addition to physical . . . | Physical wellbeing | Dimensions of wellbeing | Whole person health |
| 4 | Well-being | So well-being means to me. Like a mental, it's a mental . . . | Mental wellbeing | Dimensions of wellbeing | Whole person health |
| 4 | Well-being | (So well-being means to me) physical . . . | Physical wellbeing | Dimensions of wellbeing | Whole person health |
| 4 | Well-being | (So well-being means to me) what you eat . . . | Nutritional wellbeing | Dimensions of wellbeing | Whole person health |
| 4 | Well-being | (So well-being means to me) work life balance . . . | Feeling balanced | Feelings of wellbeing | Whole person health |
| 4 | Well-being | (So well-being means to me) Having quality friendships and relationships. | Social wellbeing | Dimensions of wellbeing | Whole person health |
| 4 | Well-being | (So well-being means to me) Hobbies, having hobbies that you can like disconnect from the world to go to kind of reground yourself. | Hobbies | Dimensions of wellbeing | Whole person health |
| 4 | Well-being | (So well-being means to me) Not losing yourself in your job because you're a functioning human outside of that. | Ways of being | Holistic wellbeing | Whole person health |
| 4 | Well-being | You know, I would say holistic well-being, it encompasses everything, you know . . . everything. | Holistic wellbeing as a multi-dimensional system | Holistic wellbeing | Whole person health |
| 4 | Well-being | (You know, I would say holistic well-being, it encompasses everything, you know) social . . . | Social wellbeing | Dimensions of wellbeing | Whole person health |
| 4 | Well-being | (You know, I would say holistic well-being, it encompasses everything, you know) emotional . . . | Emotional wellbeing | Dimensions of wellbeing | Whole person health |
| 4 | Well-being | (You know, I would say holistic well-being, it encompasses everything, you know) physical . . . | Physical wellbeing | Dimensions of wellbeing | Whole person health |
| 4 | Well-being | (You know, I would say holistic well-being, it encompasses everything, you know) spiritual relationships . . . | Spiritual wellbeing | Dimensions of wellbeing | Whole person health |
| 4 | Well-being | So holistic means not just looking at like your diet or how much you exercise, but everything, how is everything connected together? How are you functioning within that system? I take a systems approach, or a holistic approach. Not just one thing but everything together. And how is that overall doing for you in your wellbeing? | Holistic wellbeing as a multi-dimensional system | Holistic wellbeing | Whole person health |
| 4 | Well-being | I see it as mind body spirit balanced . . . |  |  |  |
| 4 | Well-being | Mind, body, spirit, and to other people. | Holistic wellbeing as a multi-dimensional system | Holistic wellbeing | Whole person health |
| 4 | Well-being | For me, energy and vitality are one of the kind of measures to tell if you're there. | Feeling energy and vitality | Feelings of wellbeing | Whole person health |
| 4 | Well-being | And, your healthy . . . | Feeling healthy | Feelings of wellbeing | Whole person health |
| 4 | Well-being | . . . your healthy balance that's connected. | Feeling balanced | Feelings of wellbeing | Whole person health |
| 4 | Well-being | I would just say I agree with everything I heard so far that it's a balance of all the different aspects in your life that maybe, some may be at a higher or lower level | Feeling balanced | Feelings of wellbeing | Whole person health |
| 4 | Well-being | . . . but overall you feel like you're getting everything you need in your day to day life. | Feeling that everything is okay | Feelings of wellbeing | Whole person health |
| 1 | Yoga principles | So honestly what comes to mind is, I think of my newer staff. That tend to have perhaps the better work life balance because they're newer, you know, out of college and perhaps, you know, fewer responsibilities at home or whatever. | Perception of generational influence on work culture | Interpersonal barriers to participating in yoga / wellbeing practices | Considerations for implementing wellbeing program |
| 1 | Yoga principles | I've been as extension 23 years from staff to now faculty and I don't have time to take a breath. Nothing mindful. I am a mom of quite a bit of kiddos also, so I mean, it's not like, oh, when I go home that I'm going to breathe as well. | Culture of busyness | System barriers to participating in yoga practices | Considerations for implementing wellbeing program |
| 1 | Yoga principles | It's yoga, it's meditation, it's that . . . So, any of that would be so fantastic. And so I think it would be literally. | Yoga practices becoming more salient | Perceptions about yoga principles | Wellbeing program perceptions |
| 1 | Yoga principles | My phone would have to remind me, something on the computer would have to pop up. And I would really have to be something I would have to. Work at incorporating. In order to see. And put it in my life. That's just really where I am right now. So I'll just that's my full on statement. | Reminders to do yoga practices / integration of yoga practices into work schedule | Suggestions for FLEX program | Wellbeing program perceptions |
| 1 | Yoga principles | Well, I make time to exercise. So like. I purposely do it at the end of the day so I put my bag in I changed clothes here at the office and I go to the gym. So I don't have time for my brain to think about. Let's go home. Ahead of time | Include specific strategies for integrating FLEX practices | Suggestions for FLEX program | Wellbeing program perceptions |
| 1 | Yoga principles | . . . and then as far as yoga I'm trying to work on my yoga thing. | Yoga practices becoming more salient | Perceptions about yoga principles | Wellbeing program perceptions |
| 1 | Yoga principles | That's that's just interesting to me as far as trying to figure out when to do that. | Reminders to do yoga practices / integration of yoga practices into work schedule | Suggestions for FLEX program | Wellbeing program perceptions |
| 1 | Yoga principles | Do a little 5 min video some mornings just to kind of prepare me for the work day, | Include short, accessible videos | Suggestions for FLEX program | Wellbeing program perceptions |
| 1 | Yoga principles | . . . but as a whole, I try to definitely do go to the gym in the afternoons just to breathe. . . And I feel like when I don't go if I've been working a lot for several weeks and I haven't been I just I don't feel like my well being is in a good place. | Movement important for feeling well | Perceptions about yoga principles | Wellbeing program perceptions |
| 1 | Yoga principles | So I almost treated like a program and just put it in my calendar. | Reminders to do yoga practices / integration of yoga practices into work schedule | Suggestions for FLEX program | Wellbeing program perceptions |
| 1 | Yoga principles | I think that people assume that because we're in the Extension or because we're agents. That we have it altogether. So I personally feel like um I'm sorry. I can't. I'm not good with names on the first time, but the first person that went, I feel like I have really a problem with balance. | Perception that agents have it altogether / are balanced is incorrect | Interpersonal barriers to participating in yoga / wellbeing practices | Considerations for implementing wellbeing program |
| 1 | Yoga principles | I actually feel like planning. Some of those things in the question is like would be a part of work in until I learned how to do it. So I think for agents to have some training on it. "What is it? How do you do it? Best ways to incorporate it." Those types of things would be beneficial.. | Desire to receive training to support implementation of personal flourishing practices | FLEX program is desirable | Wellbeing program perceptions |
| 1 | Yoga principles | Because we're so busy planning our job that I think. Doing things for ourselves sometimes becomes another . . . Because sometimes Our job is such that. Doing those things actually becomes another job to us. And so we leave them out. It's been my experience. We leave. Those things out we know about them. But we leave them out because we're trying to accomplish so many other goals. | Culture of busyness | System barriers to participating in yoga practices | Considerations for implementing wellbeing program |
| 1 | Yoga principles | . . . Sometimes I think that if agents had like training and what these things were or how to implement them that that would help them towards flourishing. | Desire to receive training to support implementation of personal flourishing practices | FLEX program is desirable | Wellbeing program perceptions |
| 1 | Yoga principles | I know for me I'm not in an area that has like a good gym to go to and one of the things that was good during COVID was access to like the live streaming of the different exercise groups that are always in person, but it's like 4 or 5 h from where I'm at to the main Extension in [Place]. So yeah, like yoga or dance or anything that you had open access to would help. Or better yet training. | Safe, accessible spaces in which to practice movement | System barriers to participating in yoga practices | Considerations for implementing wellbeing program |
| 1 | Yoga principles | I had to say I agree with, what [other participant] as well as, [other participant], it almost has to be that you have to and I started a Walk With program in January and that's what has helped me personally. Become more active and more aware of my physical wellbeing, but it has also helped me with my mental health as well. . . that in returns helps us better with our mental health and physical health. | Movement important for feeling well | Perceptions about yoga principles | Wellbeing program perceptions |
| 1 | Yoga principles | So I have to put it in my calendar. I have to, you know, make sure that I'm held accountable by my, by the work participants in my program. So we hold each other accountable to to be there. | Reminders to do yoga practices / integration of yoga practices into work schedule | Suggestions for FLEX program | Wellbeing program perceptions |
| 1 | Yoga principles | We're actually playing pickleball versus doing a Walk With program is, which is, trying to stay active . . . | Include play/prizes into programming | Suggestions for FLEX program | Wellbeing program perceptions |
| 1 | Yoga principles | I know when it comes to, um, financial, um financial health. That has been very helpful to me personally as well as, so I want to learn it because I want to. Share it with people in the community, but it's been. Beneficial to me to know as well. So. That's 1 of the domain that's been beneficial as an agent. | Financial and material stability | Key domain of flourishing | Whole person health |
| 2 | Yoga principles | I did not anticipate on having so many opinions about this. Sorry. I, what comes to mind is when we're under pressure or stress. Sometimes we handle those differently depending on if we're in the workplace or in our home environment. And so as far as breath work, I find myself with my family, with my children, having to pause and take a breath. I probably should pause and take a breath before reacting. About something where at work I feel like I'm more level headed. I don't know if that's something I should admit, but. Being able to process things a little bit easier with the clear head without the distractions of little hands tugging on me and needing things all the time, you know . . . | Yoga practices perceived as beneficial for home setting, not just work setting | Perceptions about yoga principles | Wellbeing program perceptions |
| 2 | Yoga principles | . . . so as far as breath work or pausing. I don't know that it necessarily changes the situation other than it causes us to pause and evaluate it before we react to it. So we can better process what's happening in any situation under stress in any of those domains. | Pausing / breathwork perceived as important | Perceptions about yoga principles | Wellbeing program perceptions |
| 2 | Yoga principles | Yeah, I agree with (participant) in terms of. of stopping in and, not even thinking sometimes, it's just stopping and, that, creating that pause. Helping to either think or either react differently or either just rest. But it's like, a break. In a sentence. Sometimes it's helpful, I think sometimes, it's not depending on the situation depending on on the personality. I think sometimes I can understand it better than other times. So it depends. | Pausing / breathwork perceived as important | Perceptions about yoga principles | Wellbeing program perceptions |
| 2 | Yoga principles | I'm agreeing with all of y'all. Good points [about pausing to evaluate while responding to stress]. | Pausing / breathwork perceived as important | Perceptions about yoga principles | Wellbeing program perceptions |
| 3 | Yoga principles | I like that you gave an array of different things there because I think everybody has something that works and that for similar outcomes or different capacities within that list. I mean, for me, all of those things are really crucial to my flourishing, but I don't know that they're crucial for other Extension employees' flourishing. I'm sure you know everyone kind of has their own toolkit of what works or should have their own toolkit of what works, but. I think those are all good things in the in a toolkit. | Yoga principles as part of toolkit for diverse audience to use towards their personal flourishing | Perceptions about yoga principles | Wellbeing program perceptions |
| 3 | Yoga principles | Yeah, with the thriving not just surviving like I guess I think of how our jobs are very demanding and we are pulled in lots of different directions at any given time. And so to me some of those things are. They hope to in the middle of stressful times and stuff they kind of help you to take a space and a breath and to be able to kind of calm down the physical (inaudible), the of feeling anxious or stressed especially like if you're preparing for a program or something and you're just it's like. Wow, I have so much to do and it's feeling overwhelming at the moment and I don't feel like I'm gonna get it done. | Yoga practices can provide space to thrive, not just survive | Perceptions about yoga principles | Wellbeing program perceptions |
| 3 | Yoga principles | Sometimes just taking that pause can really help you to not be just surviving. i guess is just one practical way that I think about it. | Pausing / breathwork perceived as important | Perceptions about yoga principles | Wellbeing program perceptions |
| 3 | Yoga principles | For me, I always try to take a walk at lunch. It helps me get out of the office and get fresh air and just kind of, you know, get away from everything. | Movement important for feeling well | Perceptions about yoga principles | Wellbeing program perceptions |
| 3 | Yoga principles | And so I'm glad that that's like, I don't know, supported because other people in my office do it as well. So it's kind of like this. Maybe we'll have lunch together sometimes, but for the most part it's not a big deal if you wanna, you know, eat together for 30 min in the walk for 30 min or, you know, step out during the day to get some fresh air. So I think that helps us as an office. Especially because I just now have a window but before that I didn't have a window. So it's definitely needed. | Office culture of taking breaks for movement | System-level support for participating in yoga practices | Considerations for implementing wellbeing program |
| 3 | Yoga principles | I have a very similar, similar situation where in my office it's really supported to go out and take a break and walk and take time for yourself and that's okay. But I feel like across at least our state and Virginia across our state, it's not necessarily as acceptable in other offices. That like I feel like I'm merely blessed to be in a pretty flexible office but I think even up above. In administrative, they don't necessarily uh support those breaks. They, you know, if you ask them, "hey, is it okay if I take my lunch break and walk that come back and still eat my lunch?" "Oh yeah, as long as it's with in that hour, 30 min, you know, whatever you're restricted to, that's all you got." Fortunately, I'm not in that kind of situation where it's really strict, but I can see that in other offices it might not be a conducive environment. . . But I'll also add, my colleagues and I started walking, so we would take our lunch after we ate lunch. All of us would go and walk. We have we were really fortunate to have a track behind our office. | Office culture of taking breaks for movement | System-level support for participating in yoga practices | Considerations for implementing wellbeing program |
| 3 | Yoga principles | Also, because our offices are all over the place. Some offices don't have the same opportunities to be able to go walk in a safe space. Or to be able to take advantage of being able to go outside or walk around their building or whatever. So it's it's definitely tough. | Safe, accessible spaces in which to practice movement | System barriers to participating in yoga practices | Considerations for implementing wellbeing program |
| 3 | Yoga principles | And then I see certain people who they would fight the idea like they it's like they wouldn't utilize the trainings or the opportunities that might be offered. Because That's just the way their view is on taking a mindful moment or taking an exercise break. | Resistant views to pausing / taking a break for self-care | Individual barriers to participating in yoga practices | Considerations for implementing wellbeing program |
| 3 | Yoga principles | Or they just have this mentality of like, "oh, I'm just too busy, I can't do that." So I could see like some people. Colleagues would fight the idea of given that time they wouldn't take it. | Culture of busyness | System barriers to participating in yoga practices | Considerations for implementing wellbeing program |
| 3 | Yoga principles | So we would go on a walk and we were actually going to do a poster. For, winter conference that talked about the improvement in our office as a whole because we as a team would go out and walk and just be able to talk about work, connect. It's sort of like having a walking staff meeting. Is what it was sort of like, but we get to really confide in each other and then talk about what's going on and it really strengthened our office big time just taking these these breaks which was really cool | Include specific strategies for integrating FLEX practices | Suggestions for FLEX program | Wellbeing program perceptions |
| 3 | Yoga principles | I would just add on to what everyone said about like I think there's a little bit of mixed messaging. I know when I started you know, I've had these practices for a long time. And when I was interviewed, I was asked about my ability to multitask and I said that's not a thing. Like, your brain can't do that, and I think it almost prevented me from being hired. So there's a little bit of a lack of understanding about like a culture of busyness versus being able to take time for these things. | Culture of busyness | System barriers to participating in yoga practices | Considerations for implementing wellbeing program |
| 3 | Yoga principles | And I know like we get emails and messages about dealing with burnout through like the calm app that we have access to. And we do have flexible work but it's. The problem is, well, and I don't want to say the problem is, but the problem is, is that I think there's I think these practices also aren't well understood. Because of the kind of era of McMindfulness that you can talk about them without being invested in them. So that would be why I said things the way I did the first time around, as in they're not for everyone because you can't just necessarily drop into it for a minute, but be doing 2 things at once for the rest of your day. | Yoga practices are poorly understood | System barriers to participating in yoga practices | Considerations for implementing wellbeing program |
| 3 | Yoga principles | Yeah, I definitely think boundaries on, you know, cause with our jobs it's really variable, right? We can work nights, we can work weekends, we can work all kinds of hours outside of the normal office hours. And so I, when I started my career and extension, I was the 'check my email on the weekends' kind of person and doing all these different things. And I had a coworker she was a 4H agent in my office, and she was like don't do that, like immediately like it was my first year you need to go ahead and like just decide not to. And ever since then I've had pretty strict boundaries with like I'll work from this time to this time. It's going to be on my calendar. I'm going to take my entire lunch break. I'm not going to check emails. The phone rings. It can go to voicemail. | Include setting boundaries | Suggestions for FLEX program | Wellbeing program perceptions |
| 3 | Yoga principles | And I've had other people comment on it just kind of like, you know, but I don't know. I don't know. Post-COVID or whatever this one coworker just kind of emphasizing it to me, but I'm sure it's people can't really get away with that I guess in other office is probably another state. So I'm fortunate that I can do that I guess without really getting in trouble necessarily but, yeah, I think it's definitely like a cultural shift of, a lot of. A lot of people who are just kinda like that, like always available. | Culture of busyness | System barriers to participating in yoga practices | Considerations for implementing wellbeing program |
| 3 | Yoga principles | And I remember at one of our early career conferences, there was a panel of older, older agents, more seasoned agents, who were talking about work life balance and all these things and a lot of them are repeating the same thing where once they had kids, they were really fortunate that extension was so flexible and they were able to like take more time with their kids. But before that, it was all, it seems like they were all working crazy hours and continuously, and I was just kind of sitting there like. Well, not everyone has kids, so I don't necessarily think that means that you should be working non-stop your whole career and you know it was definitely like an older generation kind of like commentary. | Perception of generational influence on work culture | Interpersonal barriers to participating in yoga / wellbeing practices | Considerations for implementing wellbeing program |
| 3 | Yoga principles | So I feel like I don't know if it's due to COVID or just kind of like changing trends, but I think mindfulness and a real work life balance is becoming more and more talked about and hopefully respected. We'll see. | Yoga practices becoming more salient | Perceptions about yoga principles | Wellbeing program perceptions |
| 3 | Yoga principles | I was going to comment on the generation differences as well. In my office we have one person who is just retired, but we have a couple of people who are over the age of 60. And just their mindset towards working, and being on the clock and being available is very different than younger folks. I think that. You know, I am now their director, and I'm, like, 35, and so I think that like I have to kind of push this mindset on them. I mean, obviously it has worked for them. I mean, obviously it has worked for them for some to some degree for some time, but you know burnout is real, and I think especially as as we get older, as they get older I just want them to be mindful of that. So it's just interesting to see how. Place out in our office. | Perception of generational influence on work culture | Interpersonal barriers to participating in yoga / wellbeing practices | Considerations for implementing wellbeing program |
| 4 | Yoga principles | You know, I am recently got feedback from a colleague of mine, a long time colleague. And then actually an administrator and they said, you know, When you did that yoga thing in that breathing and you know mindfulness activity. We thought it was kind of silly, but now we use it a lot. . . and that. They for lack of a better way of saying this are "believers" in it now. | Yoga practices becoming more salient | Perceptions about yoga principles | Wellbeing program perceptions |
| 4 | Yoga principles | And that they. I would say they experience quite a high amount of stress in their positions. Extension and also you know we all experience stress in our lives but they gave me feedback. It really they really are using it and then it has been helpful and that. . . So it was good for me to get that feedback because I, you know, I didn't know for sure. I mean, I think it's important. I think it's helpful. But to hear them in their own words. Tell me that they're using the box breathing or the mindfulness exercises. | Yoga principles as part of toolkit for diverse audience to use towards their personal flourishing | Perceptions about yoga principles | Wellbeing program perceptions |
| 4 | Yoga principles | And then I've done a lot with youth in programs and they too have come back to me and told me. How much, it's been helpful for them and how they use it. | Yoga principles as part of toolkit towards mutual flourishing / Extension programming for diverse contexts | Perceptions about yoga principles | Wellbeing program perceptions |
| 4 | Yoga principles | So from that I would say You know, I even now think (yoga principles are) more important and more useful. | Yoga practices can provide space to thrive, not just survive | Perceptions about yoga principles | Wellbeing program perceptions |
| 4 | Yoga principles | I would say I see it as another tool in my toolkit because it's something that I can pair with other evidence-based information to make sure that You know, people are having an overall experience with me in Extension. If that makes sense. So there's a lot of different organizations that maybe they're just doing one little thing but that's all they're doing. But this is a way for me to include. Some physical activity, some community building, some group dynamics with my overall presentations. And that way, you know, maybe they're not here for the nutrition. They're here for the for the yoga, but I'm able to combine those 2 things or it's the other way around that maybe they're there for nutrition, but hey, I've given you another tool that can help you kind of be mindful in other aspects of your life. So just another skills that I'm looking to add. | Yoga principles as part of toolkit towards mutual flourishing / Extension programming for diverse contexts | Perceptions about yoga principles | Wellbeing program perceptions |
| 4 | Yoga principles | I consider those modalities. And Just like, like you said, toolkit, part of your toolkit, I agree. | Yoga principles as part of toolkit for diverse audience to use towards their personal flourishing | Perceptions about yoga principles | Wellbeing program perceptions |
| 4 | Yoga principles | Cause for me because I've studied, I am a registered dietician. I do study food. But I've also worked in the food and security space and it's hard to teach nutrition to someone who is stressed. They're not gonna be receptive of the information we're trying to share, because a lot of times they already know what they need to do, but it's hard to navigate it because of what's going on in our life. So I see it going hand in hand with: Look, let's address the stress first. So you can also make a effort to do this other goal that you have. | Yoga principles as part of toolkit towards mutual flourishing / Extension programming for diverse contexts | Perceptions about yoga principles | Wellbeing program perceptions |
| 4 | Yoga principles | I see (yoga principles) as tools to help you regulate your emotions and find that center calm. | Yoga principles as part of toolkit for diverse audience to use towards their personal flourishing | Perceptions about yoga principles | Wellbeing program perceptions |
| 4 | Yoga principles | I mean, I see (yoga practices as as way to) go with flourishing as just a way of Extension employees to be able to take care of themselves too. | Yoga principles as part of toolkit for diverse audience to use towards their personal flourishing | Perceptions about yoga principles | Wellbeing program perceptions |
| 4 | Yoga principles | In addition to teaching (yoga principles) to the communities. | Yoga principles as part of toolkit towards mutual flourishing / Extension programming for diverse contexts | Perceptions about yoga principles | Wellbeing program perceptions |
| 4 | Yoga principles | Cause I know in my office I tend to be the way my office is set up. I have one of the larger offices. I have a couch and I kind of been unexpectedly dubbed the therapist. Office like folks when they go through things they they come to my office. And I would love to see them have an outlet. To kind of release some of those stresses that we, you know, I've been available to, you know, hear, but like I said, I need a way out, an outlet myself, so. I just see as needed is a part of the toolkit of balancing those emotions and the things that go on. | Yoga principles as part of toolkit for diverse audience to use towards their personal flourishing | Perceptions about yoga principles | Wellbeing program perceptions |
| 4 | Yoga principles | And so I'll be honest. I don't use it in my day to day life now. I don't know much about it, but I can see the benefit is that it's, you're not dependent on your space or what equipment you have. It's something that can be done anywhere. And you can use your surroundings and incorporate them in your activity. . . So regardless of where you are or even, you know, the age range of the people you're dealing with. Or their activity level. It's something that everyone can do and feel included. | Yoga practices perceived as accessible | Perceptions about yoga principles | Wellbeing program perceptions |
| 4 | Yoga principles | So I think (training in yoga principles) would be a really helpful tool to have if that makes sense. | Yoga principles as part of toolkit for diverse audience to use towards their personal flourishing | Perceptions about yoga principles | Wellbeing program perceptions |
| 4 | Yoga principles | I find I need all three (yoga practices) daily, and if I don't, I tend to get out of thriving. Even a day off. I definitely think it's very useful. I figure the more years I do it, then maybe it won't be so hard. | Yoga practices can provide space to thrive, not just survive | Perceptions about yoga principles | Wellbeing program perceptions |
| 4 | Yoga principles | And I think for, you know. Thriving, flourishing, moving your body and being able to breathe through stressful times or even just having that physical release of moving your body throughout the day is super important. And I think an extension where you're sitting a lot. We're in stressful situations . . . | Yoga practices can provide space to thrive, not just survive | Perceptions about yoga principles | Wellbeing program perceptions |
| 4 | Yoga principles | . . . And then we come home and that ( work stress) comes home with us. So I think (yoga principles are) like a 360 important and all, and not just the workplace, but even at home and. Kind of dido to what everyone else said. | Yoga practices perceived as beneficial for home setting, not just work setting | Perceptions about yoga principles | Wellbeing program perceptions |
| 4 | Yoga principles | Yeah, I would definitely have to agree. I think we spend a lot of time focusing on people outside of our organization and how we can benefit their health and impact them in a positive way that we tend to forget about ourselves and like. We're that same person that we're trying to tell to eat right, and move, and breathe, and function in society. And then you're like Well, I'm also that same person who is struggling with XY, and Z and trying to balance that. | Yoga practices can provide space to thrive, not just survive | Perceptions about yoga principles | Wellbeing program perceptions |
| 4 | Yoga principles | That we kinda like need an employee wellness program that focuses on those specific topics as well because we add that burden on to ourselves and then forget to focus on ourselves at the end of the day. | Need for employee wellness program | FLEX program is desirable | Wellbeing program perceptions |
| 4 | Yoga principles | And you know to kind of add to that and this is I know it's being recorded but I'm just gonna say it colleague and I were talking the other day we noticed several younger colleagues that had, you know, started in extension and within 6 months or a year you could physically see the stress on them. They'd gain weight. They were breaking out. It really got me thinking about our jobs, and how I don't think there is an institutional focus on well-being for Extension employees, not in our state, not in the nation actually so. Kind of become more and more aware of to me the need for. | Culture of busyness | System barriers to participating in yoga practices | Considerations for implementing wellbeing program |
